# Fluorine-18 Radiolabeled Single-Chain Antibody Variable Fragment 1F4 Targets α1-Subunit Gamma-Aminobutyric Acid Type A Receptors in Mice

**DOI:** 10.1021/acs.jmedchem.5c02984

**Published:** 2026-02-09

**Authors:** Ángel García de Lucas, Negar A. Samani, Olli Moisio, Luciana Kovacs, Risto Savela, Sanna L. Soini, Sami Oksanen, Jatta S. Helin, Johan Rajander, Anu J. Airaksinen, Urpo Lamminmäki, Francisco López-Picón

**Affiliations:** † Turku PET Centre, University of Turku and Turku University Hospital, FI-20520 Turku, Finland; ‡ PET Preclinical Imaging Laboratory, Turku PET Centre, 8058 University of Turku, FI20520 Turku, Finland; § Department of Life Technologies, 8058 University of Turku, FI-20520 Turku, Finland; ∥ Department of Chemistry, 8058 University of Turku, FI-20500 Turku, Finland; ⊥ VARHA, The Wellbeing Services County of Southwest Finland, FI-20520 Turku, Finland; # Integrative Physiology and Pharmacology, Institute of Biomedicine, 8058 University of Turku, FI-20520 Turku, Finland; ∇ Accelerator Laboratory, 1040 Åbo Akademi University, FI-20520 Turku, Finland

## Abstract

Gamma-aminobutyric acid type A (GABA-A) receptors are the principal inhibitory neurotransmitter receptors in the central nervous system (CNS), but their functions in the peripheral nervous system (PNS) and organs such as the heart remain poorly understood. These receptors comprise various subtypes based on subunit composition with differential brain and heart expression linked to distinct pathologies. Current positron emission tomography (PET) imaging protocols use radioligands lacking subtype specificity. To address this, we developed a PET tracer targeting the α1 subunit. The α1-specific single-chain variable fragment (scFv) 1F4 was engineered from the variable domains of monoclonal antibody (mAb) 1F4. It was efficiently ^18^F-labeled under mild conditions via biorthogonal inverse electron demand Diels–Alder (iEDDA) ligation. PET biodistribution in mice showed favorable pharmacokinetics for [^18^F]­F-Tz-TCO-scFv 1F4 with specific α1 subunit binding in the brain, heart, and lungs. This tracer promises to evaluate GABA-A α1 distribution and expression in peripheral organs, particularly the heart.

## Graphical abstract

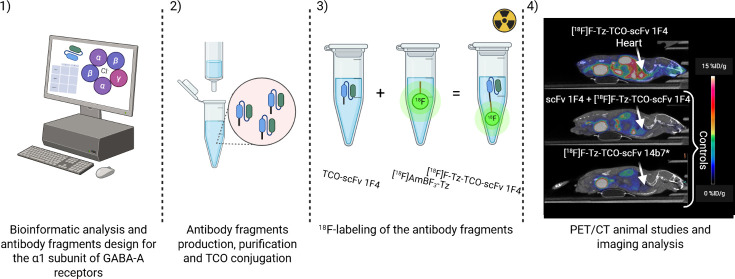

## Introduction

Gamma-aminobutyric acid type A (GABA-A) receptors are the principal inhibitory neurotransmitter receptors in the central nervous system (CNS), yet their presence in the peripheral nervous system (PNS) and non-neural tissues—such as the heart—remains underexplored.^1,2^ GABA-A receptors are pentameric ion channels composed of subunits selected from 19 types: six α (α1–6), three β (β1–3), three γ (γ1–3), three ρ (ρ1–3), and one each of δ, ε, π, and θ.^3^ Receptor subtypes are classified by their α subunits.^4^ GABA-A receptors containing α1, α2, α3, or α5 subunits are benzodiazepine-sensitive, whereas those with α4 or α6 subunits are not.^5^ Distinct α subunits confer specific pharmacological profiles with α1 subunit associated with sedation and α2 subunit, and possibly α3 subunit, associated with anxiolysis.^6^ Most GABA-A receptors are αβγ heteropentamers. In the brain, the α1β2γ2 isoform predominates and accounts for approximately 60% of GABA-A receptors.^5^

Beyond the CNS, the GABAergic system is distributed throughout the PNS and non-neural tissues, yet its regional diversity and function remain comparatively understudied. Expression outside the brain is broad but tissue-specific. Mouse tissue immunoblotting has revealed organ-selective GABA-A receptor expression with the α1 subunit markedly enriched in the heart and bladder and present at lower abundance in the stomach, lung, kidney, and liver. There is also evidence for stress-induced plasticity of peripheral GABA-A receptor expression.^2^ Additional studies in rodents, particularly rats, have identified GABA-A receptors in the adrenal gland, ovary, testis, placenta, uterus, and small intestine and with widespread expression throughout the enteric nervous system.^7,8^ Functional GABA-A receptors are present on immune cells; α1 subunit has been detected in murine peritoneal macrophages and in human and rodent T lymphocytes.^9–11^ Pancreatic β-cells also express functional GABA-A receptors that depolarize the cell and modulate insulin secretion.^12^

Differential GABA-A receptor expression in the brain is implicated in disorders such as affective syndromes, schizophrenia, epilepsy, Down syndrome, and autism.^13–15^ Beyond the CNS, these receptors also play key roles in cardiovascular pathophysiology. For example, GABA-A receptors contribute to ventricular arrhythmias after an acute myocardial infarction. Functional GABAergic signaling, including GABA-A receptors, has been identified in sympathetic neurons of the superior cervical ganglion, where its activation suppresses sympathetic activity and confers cardioprotection.^16^ Another cardiac pathology related to GABA-A receptors through immune cells is pressure-overload hypertrophy (POH). In particular, GABA-A receptor activity in cardiac monocytes/macrophages influences myocardial hypertrophy and fibrosis following POH, and receptor blockade has shown potential in mitigating pressure overload-induced heart failure.^17^ A better understanding of GABA-A receptor subtype expression and distribution in the heart could foster the development of targeted therapies for ventricular arrhythmias and myocardial fibrosis using GABA-A receptor-based pharmacological interventions.

Positron emission tomography (PET) imaging of GABA-A receptors has relied primarily on nonselective benzodiazepine-site radioligands such as [^11^C]­flumazenil or [^18^F]­flumazenil (Figure 1A,B, respectively), which bind to α1, α2, α3, and α5 subunits with high affinity but show markedly lower affinity for α4 or α6 subunits. [^11^C]­Ro15-4513 (Figure 1C), an inverse agonist, binds all α subunits, showing the highest affinity for α5.^18^ Attempts to achieve true *in vivo* selectivity remain challenging. For example, [^11^C]­ADO was developed for α2/α3 subunit selectivity (Figure 1D) but did not demonstrate selective binding *in vivo*.^19^ Although these neuroimaging ligand studies permit reliable quantification of receptor densities, the diversity of GABA-A receptors and differential distribution in the brain presents an interpretative challenge.^20,21^

**Figure 1:**
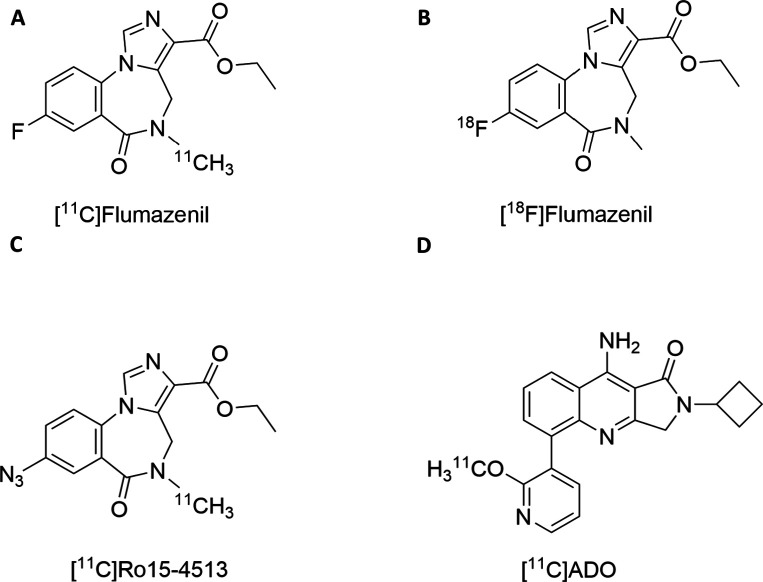
GABA-A receptor PET tracers. (A) [^11^C]­flumazenil, which exhibits preferential binding to α1, α2, α3, and α5 subunits compared with α4 and α6 subunits, (B) [^18^F]­flumazenil, demonstrating the same subunit selectivity profile as [^11^C]­flumazenil, (C) [^11^C]­Ro15-4513, showing markedly higher selectivity for the α5 subunit relative to α1, α2, α3, α4, and α6 subunits, and (D) [^11^C]­ADO, reported to preferentially bind α2 and α3 subunits; however, its subunit selectivity has not yet been conclusively established.

Immuno-positron emission tomography (immunoPET) involves tracking and quantification of radiolabeled monoclonal antibodies (mAbs), their engineered fragments, and peptides *in vivo*. This approach has demonstrated success in developing PET probes with high specificity for a range of oncologic and CNS targets.^22^ As demonstrated by a number of studies, antibody-based imaging provides a specific, sensitive, and noninvasive means for molecular detection of the cell surface proteins *in vivo* (e.g., ion channels), which aids diagnosis, prognosis, therapy selection, and treatment monitoring for various diseases.^23^ Notably, smaller antibody formats—such as single-chain variable fragments (scFvs, ∼25 kDa) lacking the Fragment crystallizable (Fc) domain—offer improved pharmacokinetics, including faster blood clearance and reduced background signal, compared to full-length antibodies.^24^ These properties enhance their utility as PET ligands, particularly for targets requiring rapid imaging or high contrast.

Here, we describe the design, fluorine-18 radiolabeling, and *in vivo* evaluation of an antibody fragment, scFv 1F4 (**1**), that targets the α1 subunit of GABA-A receptors. To assess its feasibility for α1 subunit-specific GABA-A receptor PET imaging, tissue uptake in mice was evaluated. Specificity of **1** binding was demonstrated by blocked and negative control groups and further validated by *ex vivo* biodistribution, autoradiography, and immunofluorescence studies.

## Results

### Rationale for the 1F4 Antibody Fragment Selection

mAb 1F4 was chosen for the PET tracer development based on cryogenic electron microscopy (cryo-EM) showing the antibody variable domains in complex with the human α1β2γ2 GABA-A receptor.^25,26^ To assess the suitability of mAb 1F4 for PET studies in mice, we evaluated its capacity to specifically bind the mouse α1 subunit through structural and sequence analysis. The cryo-structures of the mAb 1F4 in complex with the human GABA-A receptor (PBD: 6D6T and 6X3Z) showed that the antibody fragment interacts exclusively with the α1 subunit in the GABA-A receptor (Figure 2A). More detailed contact analysis identified a total of ten α1 subunit residues making direct interactions with the antibody; eight are shown in Figure 2B. Multiple-sequence alignment of the human α1 and mouse α1−α6 protein sequences of GABA-A receptor showed that the majority of the residues involved in specific interactions are nonconserved among α subunits but are conserved between the human and mouse α1 subunit (Figure 2C). Notably, four of the ten contact residues (Glu^170^, Glu^174^, Ala^176^, and Asp^199^) are unique to the α1 subunit. The specific interactions are listed in more detail in Supporting Information Table S1. Hence, the protein interaction analysis strongly supports the specificity of mAb 1F4 against the α1 subunit in the mouse.

**Figure 2:**
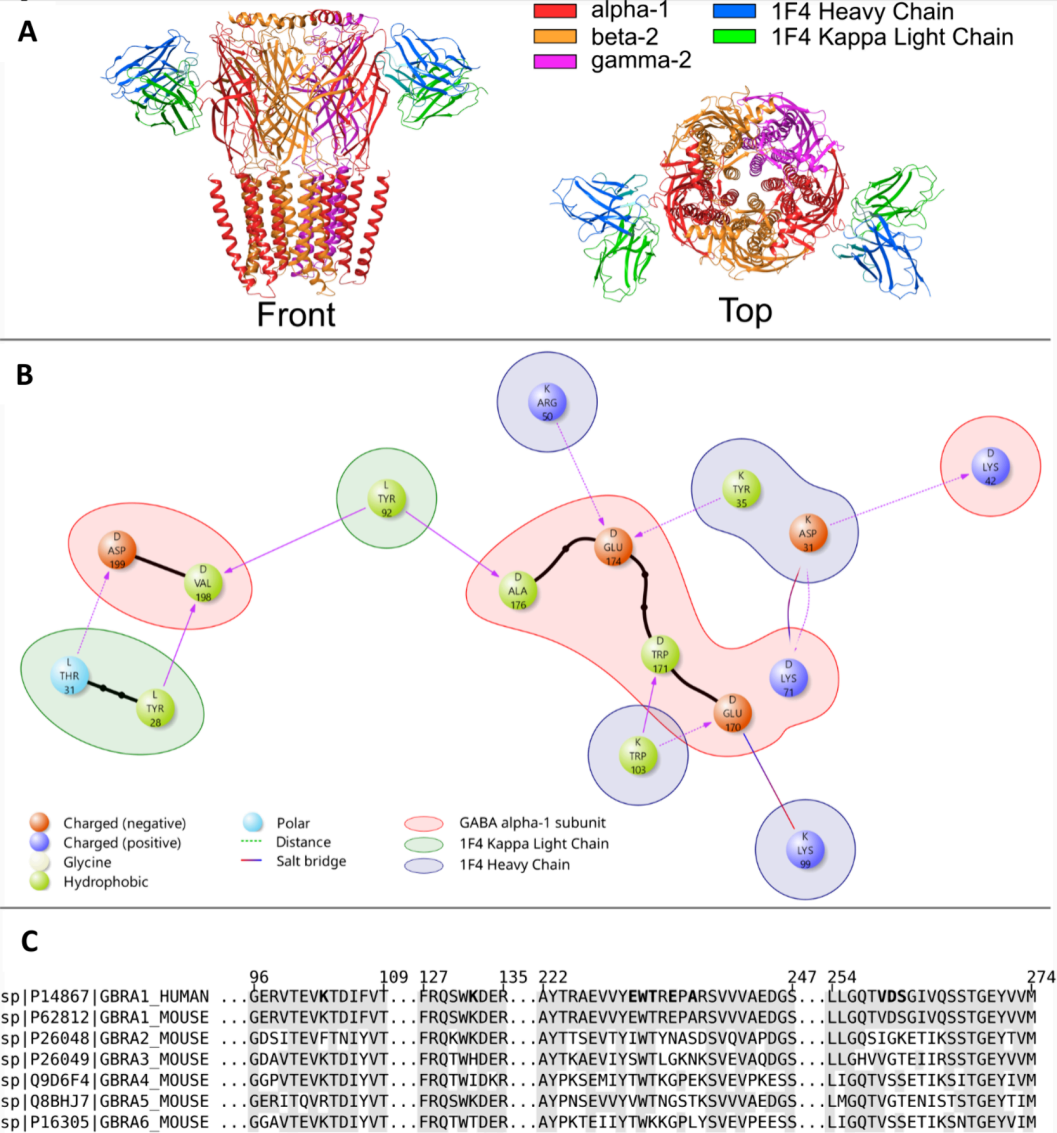
Structural visualization and interaction analysis of the GABA-A receptor with fragment antigen-binding region (Fab) 1F4. (A) Cryo-EM complex (PDB 6X3Z) illustrating Fab 1F4 bound to the α1 subunit of the human α1β2γ2 GABA-A receptor (α1, red; β2, orange; γ2, purple; heavy chain 1F4, blue; and κ light chain 1F4, green). (B) Interaction diagram for 6X3Z generated with Protein Interaction Analysis with Maestro BioLuminate software, showing residues forming specific contacts between Fab 1F4 (blue/green backgrounds) and α1 subunit (red background). (C) Multiple sequence alignment of human α1 subunit and mouse α1−α6 subunits; regions homologous to human α1 subunit are shaded, and contact residues are bolded. Most contacts lie in sequence regions that are nonconserved across α subunits.

### Antibody Fragment Production

An antibody fragment **1** was derived from mAb 1F4 by introducing a 13-amino acid peptide linker between the variable light and heavy domains. Antibody fragments **1** and the negative control anti-antrax toxin scFv 14b7* (**2**) were recombinantly expressed in Expi293f mammalian cells, purified by immobilized-metal affinity chromatography (IMAC), and analyzed with SDS-PAGE to assess size and purity. Both scFvs appeared as single predominant bands at the expected molecular weights (28.39 kDa for **1**, and 29.43 kDa for **2**; Figure S1A, Supporting Information).

Antibody fragments were purified by using size exclusion chromatography (SEC). The final isolated amounts of **1** and **2** were 23.3 ± 8.6 mg (8.5 ± 2.5 mg/mL; *n* = 2) and 0.4 mg (1.01 mg/mL; *n* = 1), respectively. SEC analysis of the purified fragments showed a symmetric main peak with a minor additional peak eluting in front of the main peak caused by **1** and **2** dimers (Figure S1B, Supporting Information).

### Electrophysiological Testing of scFv 1F4 (**1**) on GABA-A Receptors

The whole-cell patch-clamp technique was used to investigate whether **1** modulates GABA-A receptor function *in vitro*. GABA-evoked currents were recorded from WSS-1 cells expressing α1β3γ2 GABA-A receptors. Application of 10 μM GABA for 1 min at −60 mV induced inward currents (Figure S2A, Supporting Information). Treatment with 1 μM **1** for 3–4 min did not significantly change GABA-evoked currents (242 ± 52 pA baseline vs 230 ± 45 pA post-treatment; *n* = 15; *P* > 0.05) (Figure S2B, Supporting Information). Likewise, commercial mAb 1F4 showed no effect (218 ± 72 pA baseline vs 209 ± 56 pA post-treatment; *n* = 5; *P* > 0.05) (Figure S2C, Supporting Information). These data suggest that neither **1** nor mAb 1F4 modulates α1 subunit-containing GABA-A receptor currents under the conditions tested.

### AmBF_3_-Tetrazine (**3**) Synthesis and ^18^F-Radiolabeling

Alkylammoniomethyltrifluoroborate tetrazine, AmBF_3_-Tz (**3**), was synthesized and radiolabeled using a modified protocol adapted from a prior method^27^ with optimizations. Radiolabeling was achieved in a single step through ^19^F/^18^F isotope exchange (IEX) by heating the **3** precursor at 85 °C for 10 min (Figure 3A). Instead of the conventional 0.9% NaCl solution, a pyridazine-HCl buffer (pH 2.0–2.2) was used both to elute [^18^F]­fluoride and as the reaction medium, consistent with earlier strategies.^27,28^ To accommodate the low reaction volume required for efficient IEX radiolabeling, an integrated setup was implemented in which an HPLC injector was coupled to the radiosynthesis module for controlled [^18^F]­fluoride trapping and elution. The injector-module coupling, together with a custom anion-exchange cartridge (AEC) packed with AG 1-X8 resin, enabled delivery of adequately concentrated [^18^F]­fluoride directly into the reaction vessel using microliter volumes of eluent and eliminated the need for azeotropic drying.

**Figure 3:**
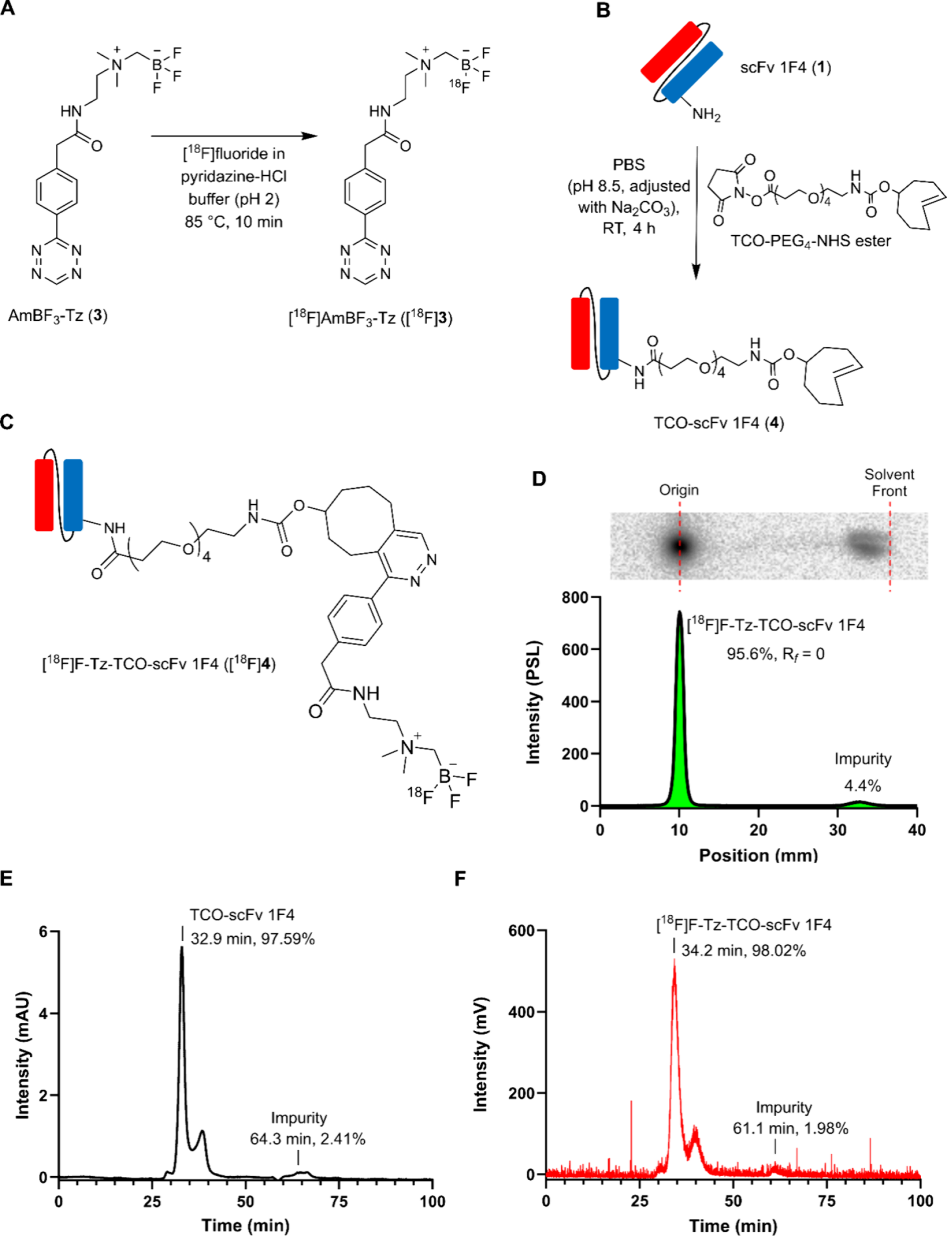
Summary of radiosynthesis, TCO-modification, and quality control (QC) of radiotracers. (A) [^18^F]­AmBF_3_-Tz ([^18^F]**3**) radiosynthesis via ^19^F/^18^F isotope exchange (IEX), (B) reaction scheme of scFv 1F4 (**1**) TCO-modification with TCO-PEG_4_-NHS, (C) chemical structure of [^18^F]­F-Tz-TCO-scFv 1F4 ([^18^F]**4**), (D) representative radio-TLC chromatogram of purified [^18^F]**4**, (E) representative UV SEC-HPLC chromatogram of TCO-scFv 1F4 (**4**) (280 nm), and (F) representative SEC-HPLC radiochromatogram of purified [^18^F]**4**.

The isolated [^18^F]­AmBF_3_-Tz ([^18^F]**3**) showed good radiochemical purity (RCP) with values of 98.4 ± 1.6% determined by radio-TLC and 96.6 ± 2.3% determined by radio-HPLC (*n* = 8; Supporting Information Figure S3). The radiochemical yield (RCY), decay-corrected to the start of the synthesis (SOS), was calculated to be 15.37 ± 6.41% (*n* = 8), and the molar activity (*A*_m_) at the end of the synthesis (EOS) was determined to be 24.6 ± 15.4 GBq/μmol (*n* = 5).

### TCO Modification and *in Vitro* Radiolabeling of Antibody Fragments

Antibody fragments **1** and **2** were functionalized by introducing *trans*-cyclooctene (TCO) groups into accessible lysine residues. For TCO modification, antibody fragments were incubated with a 15-fold molar excess of TCO-PEG_4_-NHS ester for 4 h at room temperature (RT) (Figure 3B) and purified using a 10 kDa Amicon centrifugal filter with phosphate-buffered saline (PBS) as the eluent. The purity of the TCO-conjugates was confirmed by SEC-HPLC. Additionally, mass spectrometry verified the TCO-scFv 1F4 (**4**) identity and revealed an average of 1.9 TCOs per **1** (Figure S4, Supporting Information).

Modified antibody fragment **4** was radiolabeled by [^18^F]**3** for 10 min at RT and purified by using a PD MiniTrap G-25 column. The resulting [^18^F]­F-Tz-TCO-scFv 1F4 ([^18^F]**4**) (Figure 3C) was obtained in high RCP of ≥95% as determined by radio-SEC-HPLC and radio-TLC (Figure 3D–F). The decay-corrected RCY was 45.4 ± 25.6% (*n* = 5), calculated relative to the [^18^F]**3** EOS activity and with an *A*_m_ of 23 ± 14 GBq/μmol at EOS (*n* = 6). Similarly, antibody fragment **2** was TCO-modified to give TCO-scFv 14b7*­(**5**) and radiolabeled using the same procedure, yielding [^18^F]­F-Tz-TCO-scFv 14b7* ([^18^F]**5**) with a RCY of 44.9% (*n* = 1), RCP > 96%, and *A*_m_ of 64.65 GBq/μmol (*n* = 1) at EOS (Figure S5, Supporting Information).

### Targeted PET/CT Imaging and *ex Vivo* Biodistribution

To evaluate target binding specificity and biodistribution of [^18^F]**4**, PET imaging was performed in C57BL/6J mice following intravenous (i.v.) injection of [^18^F]**4** (3.71 ± 0.19 MBq, 3.79 ± 0.80 μg), the negative control tracer [^18^F]**5** (3.33 ± 0.20 MBq, 2.31 ± 0.50 μg), and a blocking condition in which unlabeled antibody fragment **1** (50 μg, i.v.) was administered 1 h prior to [^18^F]**4** (3.81 ± 0.05 MBq, 3.68 ± 0.54 μg). PET imaging was performed on all groups (*n* = 4 per group) with a 30 min static acquisition window starting 90 min postradiotracer injection (Figure 4A). Heart uptake of [^18^F]**4** was significantly higher (7.75 ± 1.46%ID/g) than that of [^18^F]**5** (2.53 ± 0.37%ID/g, *P* < 0.05) (Figure 4B). Preadministration of unlabeled antibody fragment **1** resulted in a significant reduction in heart uptake (4.66 ± 0.32%ID/g, *P* < 0.05). Tracer uptake in the brain and lungs was also significantly higher with [^18^F]**4** (0.71 ± 0.2 and 4.58 ± 1.56%ID/g, respectively) compared to [^18^F]**5** (0.29 ± 0.08 and 2.01 ± 0.26%ID/g, respectively; *P* < 0.05 for both). Gallbladder uptake increased substantially in the blocking condition (49.6 ± 4.04%ID/g) compared to the unblocked [^18^F]**4** group (25.87 ± 9.88%ID/g, *P* < 0.05), suggesting enhanced hepatobiliary clearance of the unbound tracer.

**Figure 4:**
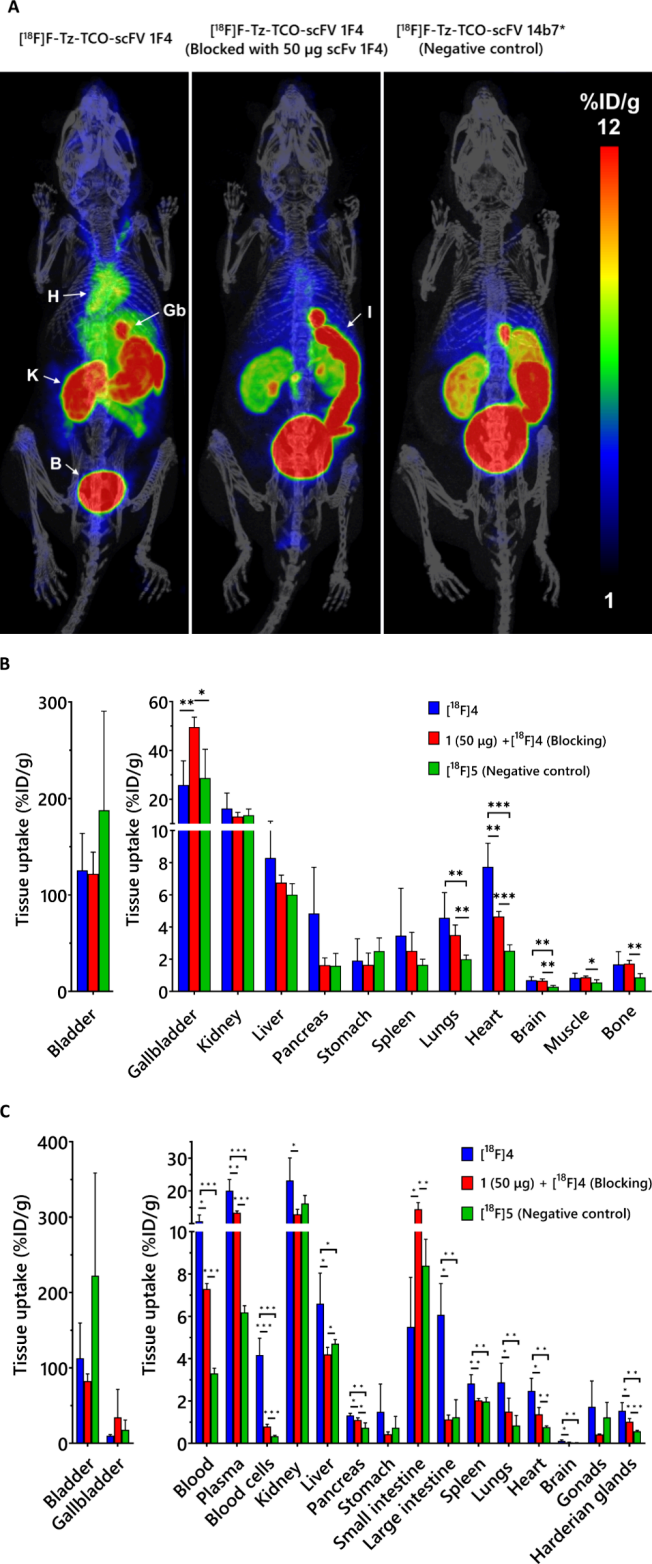
Evaluation of [^18^F]­F-Tz-TCO-scFv 1F4 ([^18^F]**4**) binding to the α1 subunit of GABA-A receptors in mice. (A) Representative PET/CT whole-body maximum-intensity projection coronal images of mice (*t* = 90–120 min) after i.v. injection of the antibody fragments (H: heart; K: kidney; B: bladder; Gb: gallbladder, I: intenstine). The conditions represented in the images from left to right are [^18^F]**4** (*n* = 4), scFv 1F4 (**1**) (50 μg, 60 min before the tracer) + [^18^F]**4** (*n* = 4), and [^18^F]­F-Tz-TCO-scFv 14b7* ([^18^F]**5**) (*n* = 4). (B) PET quantification of organ radioactivity (%ID/g) from a static scan at 90–120 min p.i. of the tracer (*n* = 4/group). (C) *Ex vivo* biodistribution (%ID/g) measured at 120 min (*n* = 4/group). Data analyzed by nonparametric multiple *t* test (*P < 0.05, **P < 0.01, and ***P < 0.001).

*Ex vivo* biodistribution analysis largely corroborated the PET imaging results, confirming significantly higher tracer uptake in the heart for [^18^F]**4** (2.48 ± 0.59%ID/g) compared with both [^18^F]**5** and the blocking group (0.77 ± 0.06 and 1.37 ± 0.32, respectively; *P* < 0.05 for both; Figure 4C). Notably, *ex vivo* analysis also revealed significantly greater accumulation of [^18^F]**4** in the brain and lungs compared to both the negative control tracer and the blocking group (Table S2, Supporting Information; *P* < 0.05), whereas PET imaging revealed significant differences only relative to the negative control (Figure 4B). Plasma radioactivity was consistently around twice that of whole blood, indicating low blood cell binding. Notably, blood cell uptake was markedly lower in both the blocking (0.80 ± 10%ID/g) and negative control (0.34 ± 0.04%ID/g) groups, representing an >80% decrease compared to [^18^F]**4** (4.17 ± 0.79%ID/g, *P* < 0.05). Several other α1-containing GABA-A receptors expressing tissues also showed significantly higher uptake with [^18^F]**4** compared to the blocking and negative control groups, notably the pancreas and liver (Table S2, Supporting Information; *P* < 0.05). Kidney uptake was significantly lower only in the blocking group (*P* < 0.05), while bladder uptake did not differ significantly across groups.

Furthermore, to evaluate the impact of the carrier competition on the α1 subunit of GABA-A receptor binding, mice were injected with either high- or low-*A*_m_ formulations of [^18^F]**4** (high: 3.76 ± 0.28 MBq, 6.62 ± 2.90 μg, *n* = 8; low: 2.57 ± 0.68 MBq, 12.08 ± 2.37 μg, *n* = 6). Imaging was conducted at 210 min postinjection with a 30 min static acquisition. The high *A*_m_ group showed significantly higher uptake in the heart, lungs, and brain than the low *A*_m_ group (*P* < 0.05), indicating competition for the α1 subunit of GABA-A receptor binding sites when low *A*_m_ tracer was administered (Figure 5A). *Ex vivo* biodistribution confirmed these results (Figure 5B and Table S3, Supporting Information).

**Figure 5:**
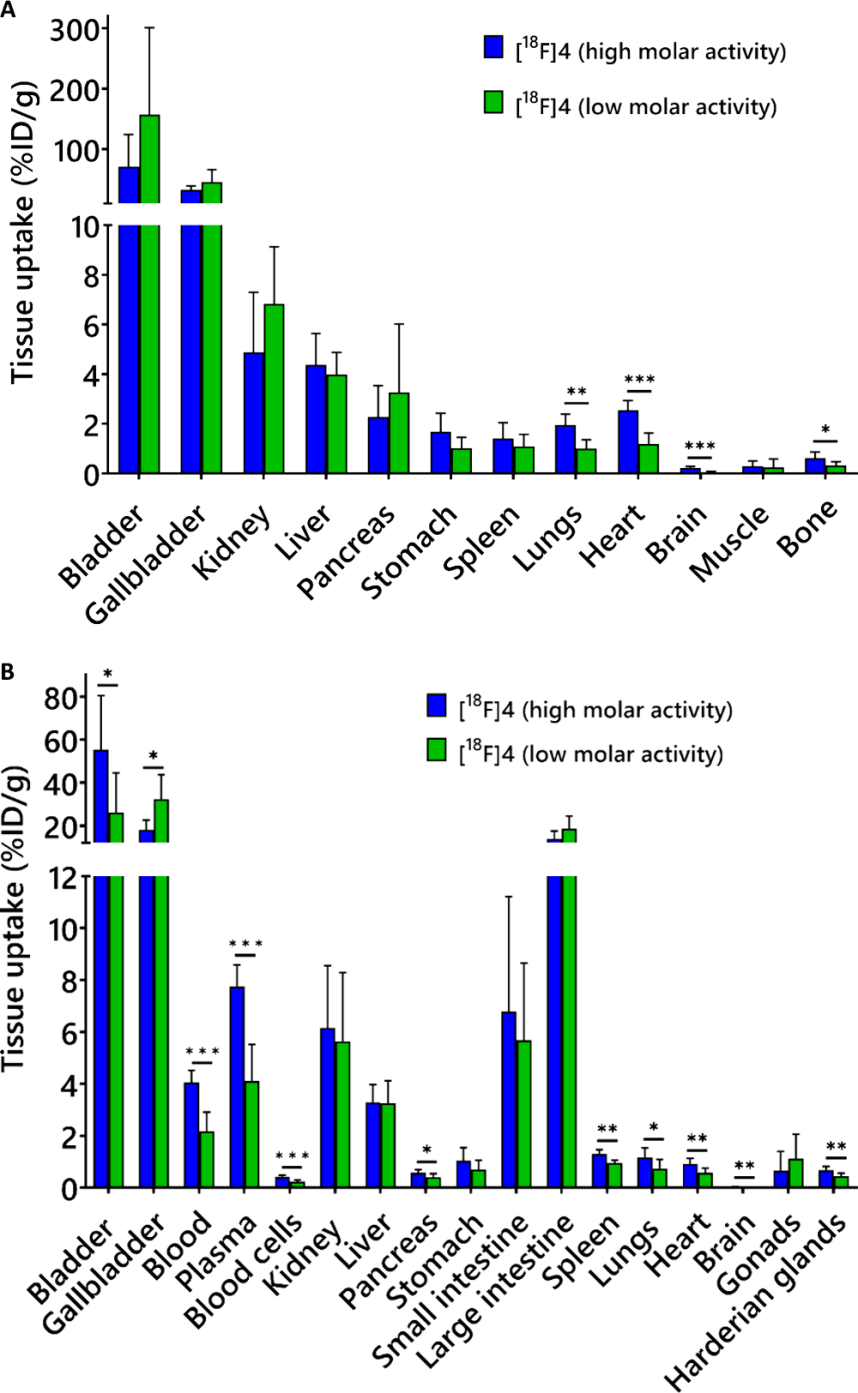
Impact of carrier on [^18^F]­F-Tz-TCO-scFv 1F4 ([^18^F]**4**) biodistribution in mice evaluated after i.v. injection of the tracer with high (injected dose 6.62 ± 2.90 μg, *n* = 8) or low (injected dose 12.08 ± 2.37 μg, *n* = 6) molar activity. (A) PET quantification of organ radioactivities (%ID/g) in both conditions from static scan at 210–240 min p.i. tracer (*n* = 6–8/group). (B) *Ex vivo* biodistribution (%ID/g) measured at 4 h (*n* = 6–8/group). Data analyzed by nonparametric multiple *t* test (*P < 0.05, **P < 0.01, and ***P < 0.001).

### Pretargeted PET/CT Imaging and *ex Vivo* Biodistribution

In the pretargeted PET imaging study, mice were intravenously injected with **4** (10 μg) 60 min prior to [^18^F]**3** injection (3.18 ± 1.09 MBq i.v.). Mice were imaged either dynamically for 60 min immediately after the tracer injection (*n* = 8) or statically for 30 min starting at 90 min after the tracer injection (*n* = 4). Control animals received only [^18^F]**3** (3.25 ± 1.11 MBq) and were imaged at identical time points (*n* = 4 for both groups) to assess nonspecific uptake of the tracer (Figure 6A).

**Figure 6:**
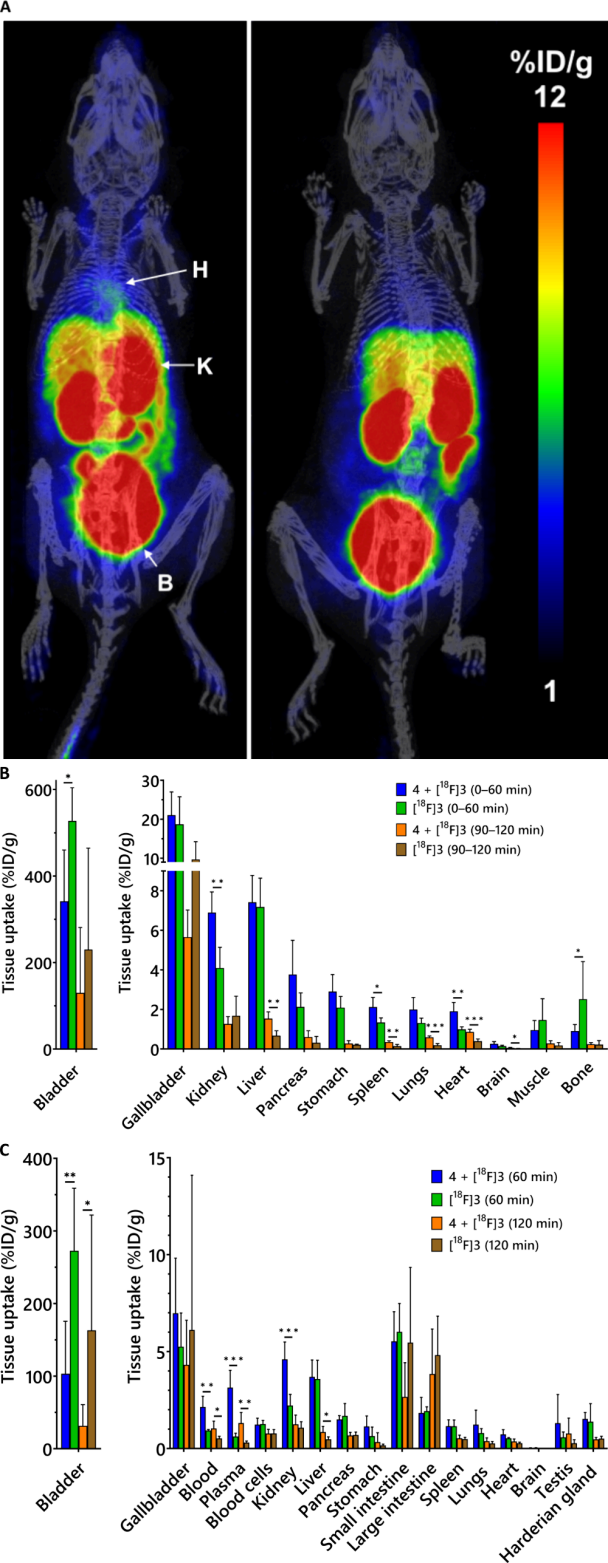
Pretargeted biodistribution studies. (A) Representative PET/CT whole-body maximum-intensity projection coronal images of mice (0–60 min) in pretargeted conditions (left) and [^18^F]­AmBF_3_-Tz ([^18^F]**3**) alone (right). For pretargeted PET imaging, TCO-scFv 1F4 (**4**) (10 μg, i.v.) was administered 60 min prior to [^18^F]**3** (H: heart; K: kidney; B: bladder). (B) PET quantification of organ radioactivity (%ID/g) at 0–60 min and 90–120 min p.i. of the tracer (*n* = 4–8/group). (C) *Ex vivo* biodistribution (%ID/g) in both conditions measured at 60 and 120 min after injection (*n* = 4–8/group). Data analyzed by nonparametric multiple *t* test (*P < 0.05, **P < 0.01, and ***P < 0.001).

Quantitative analysis revealed significantly higher heart uptake in the pretargeted group compared to control (1.91 ± 0.45 at 0–60 min and 0.86 ± 0.14 ID/g at 90–120 min vs 0.99 ± 0.13 and 0.39 ± 0.12%ID/g, respectively; *P* < 0.05), confirming successful *in vivo* ligation and specific radiotracer localization (Figure 6B). However, heart uptake with the pretargeted strategy remained lower than that achieved with the direct targeting using [^18^F]**4** (7.75 ± 1.46%ID/g at 90–120 min postinjection).

At 90–120 min postinjection of the tracer, significantly increased uptake was observed in the brain (0.06 ± 0.03 vs 0.02 ± 0.01%ID/g), lungs (0.59 ± 0.07 vs 0.19 ± 0.08%ID/g), and liver (1.54 ± 0.34 vs 0.68 ± 0.25%ID/g) in the pretargeted group relative to control (*P* < 0.05). Despite reaching statistical significance, absolute uptake values in these tissues remained low. No significant differences were noted at the 0–60 min time point, likely due to residual circulating [^18^F]**3** masking the α1 subunit-specific binding.

The spleen exhibited significantly higher uptake in the pretargeted group at both 0–60 min and 90–120 min (2.12 ± 0.49 and 0.35 ± 0.07%ID/g) compared to the control (1.34 ± 0.23 and 0.15 ± 0.09%ID/g; *P* < 0.05).

*Ex vivo* biodistribution studies failed to support the PET volume of interest (VOI) analysis showing no statistically significant differences in the radiotracer uptake in the heart, brain, or lungs between the pretargeted and control groups (Figure 6C and Table S4, Supporting Information). This discrepancy may be attributed to residual blood radioactivity contributing to the PET signal. Supporting this hypothesis, significantly higher uptake was observed in both whole blood and plasma under pretargeting conditions at 0–60 min and 90–120 min (2.14 ± 0.55 and 1.04 ± 0.37 in blood; 3.15 ± 0.89 and 1.31 ± 0.54%ID/g in plasma, respectively) compared to mice receiving [^18^F]**3** alone (0.93 ± 0.06 and 0.53 ± 0.11%ID/g in blood; 0.62 ± 0.17 and 0.31 ± 0.08%ID/g in plasma; *P* < 0.05).

### Autoradiography and Immunofluorescence Studies

*Ex vivo* autoradiographic analysis of cryosections from the heart, brain, and pancreas harvested 120 min after [^18^F]**4** injection confirmed specific binding of the tracer α1 subunit of GABA-A receptors. In the heart, [^18^F]**4** showed a high specific signal in atrial and other regions (Figure 7A) and was effectively blocked by *in vivo* administration of **1** (50 μg, 60 min prior to the tracer injection; Figure 7B). No specific signal was observed with [^18^F]**5** in the heart (Figure 7C). Immunofluorescence with a commercially available rabbit recombinant monoclonal anti-α1 subunit of GABA-A receptor antibody 1F4 confirmed colocalization of the α1 subunit expression with the observed high density autoradiography regions in the heart (Figure 7F). Additionally, successful blocking of the commercial mAb 1F4 binding with **1** further demonstrated the specificity of binding of **1** to the α1 subunit.

**Figure 7:**
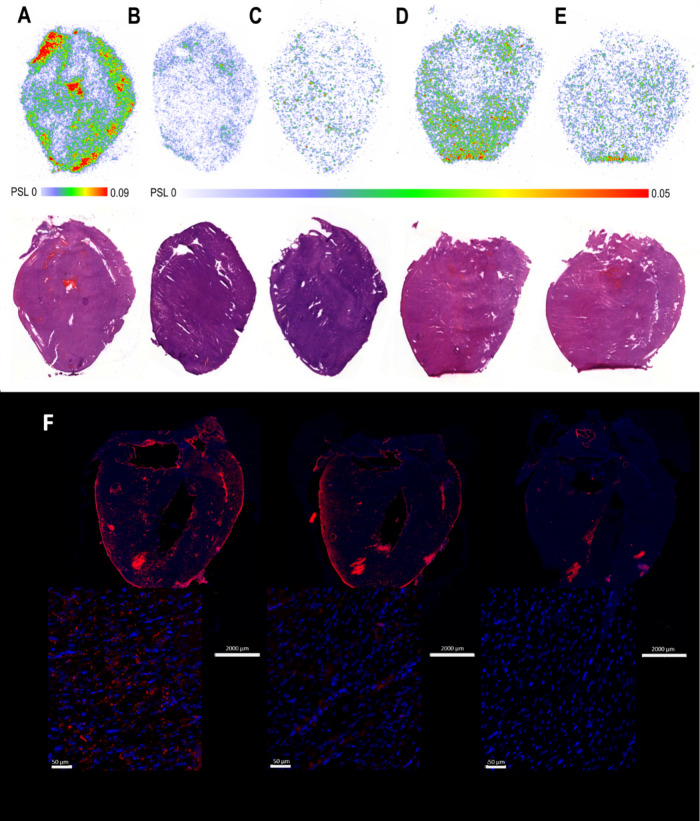
Autoradiography, hematoxylin and eosin (H&E) staining, and immunofluorescence of the heart: (A) *Ex vivo* autoradiography of [^18^F]­F-Tz-TCO-scFv 1F4 ([^18^F]**4)** binding in the heart at 120 min (top image) and H&E heart (bottom image), (B) radiolabeled antibody fragment [^18^F]**4** binding at 120 min after blocking with scFv 1F4 (**1**) (50 μg, 60 min prior to the tracer) (top image) and H&E heart (bottom image), (C) [^18^F]­F-Tz-TCO-scFv 14b7* ([^18^F]**5**) at 120 min (top image) and H&E heart (bottom image), (D) *ex vivo* autoradiography after the pretargeted strategy (10 μg of TCO-scFv 1F4 (**4**) injected 60 min prior to [^18^F]­AmBF_3_-Tz ([^18^F]**3**); heart harvested 60 min after the tracer injection) (top image) and H&E heart (bottom image), (E) radiolabeled compound [^18^F]**3** alone at 60 min (top image) and H&E heart (bottom image), and (F) immunofluorescence of the heart sections stained with mAb 1F4 and Alexa Fluor 568-conjugated secondary antibody (left), after blocking with antibody fragment **1** (middle), and with secondary antibody alone (right); nuclei were labeled with DAPI (blue). Photostimulated luminescence (PSL); maximum and minimum values indicated.

Radioactivity signal in the brain and pancreas autoradiography was lower than in the heart but almost completely blocked by antibody fragment **1** (Figures 8A,B and 9A,B). In the brain, the radiolabeled antibody fragment [^18^F]**4** showed binding primarily localized in the cerebellum and cortex (Figure 7A). In the pancreas, more intense focal dot-like signals were observed, which were absent in the blocking group and [^18^F]**5** treated animals (Figure 9A–C). The analysis of the heart and pancreas from pretargeted animals confirmed specific binding in the selected organs but with lower overall signal intensity than that observed for [^18^F]**4** (Figures 7D,E and 9D).

**Figure 8:**
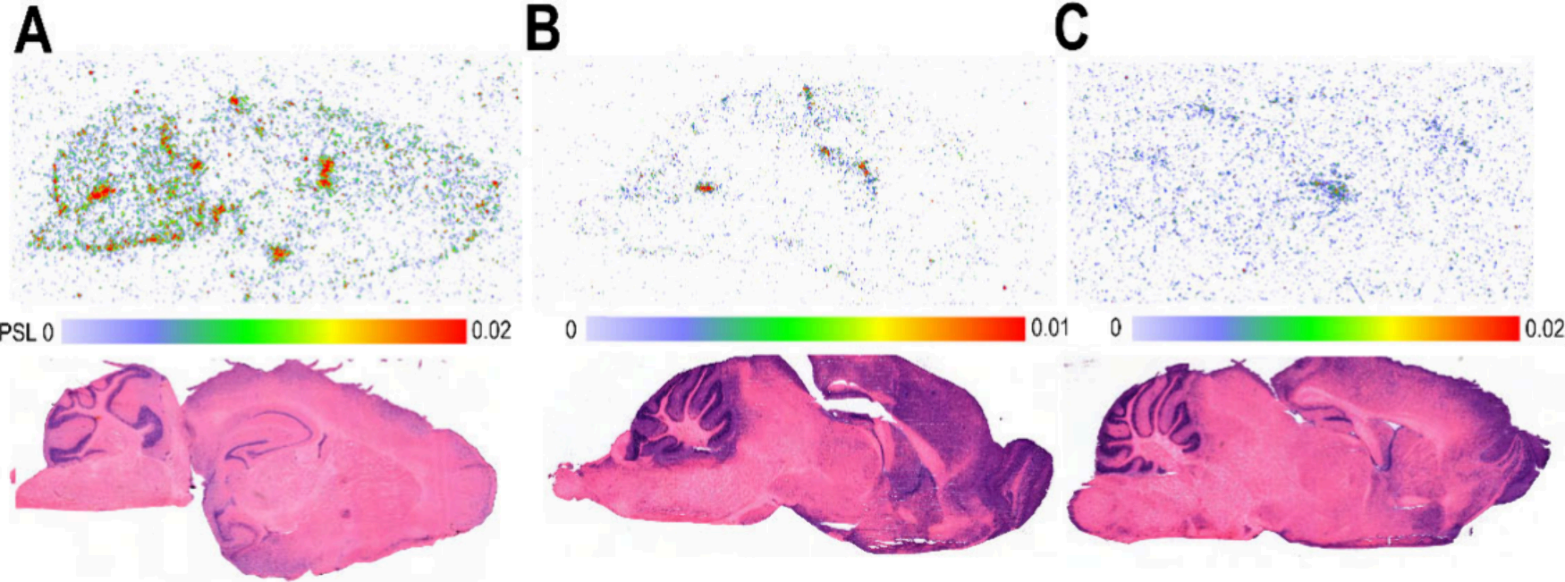
Representative *ex vivo* autoradiography and hematoxylin and eosin (H&E) images of the brain at 120 min after tracer injection. (A) Radioactivity distribution in the brain after i.v. injection of [^18^F]­F-Tz-TCO-scFv 1F4 ([^18^F]**4**) (top image) and H&E heart (bottom image), (B) after blocking with scFv 1F4 (**1**) (50 μg, 60 min prior to the tracer) (top image) and H&E heart (bottom image), and (C) after injection of [^18^F]­F-Tz-TCO-scFv 14b7* ([^18^F]**5**) (top image) and H&E heart (bottom image). Photostimulated luminescence (PSL); maximum and minimum values indicated.

**Figure 9:**
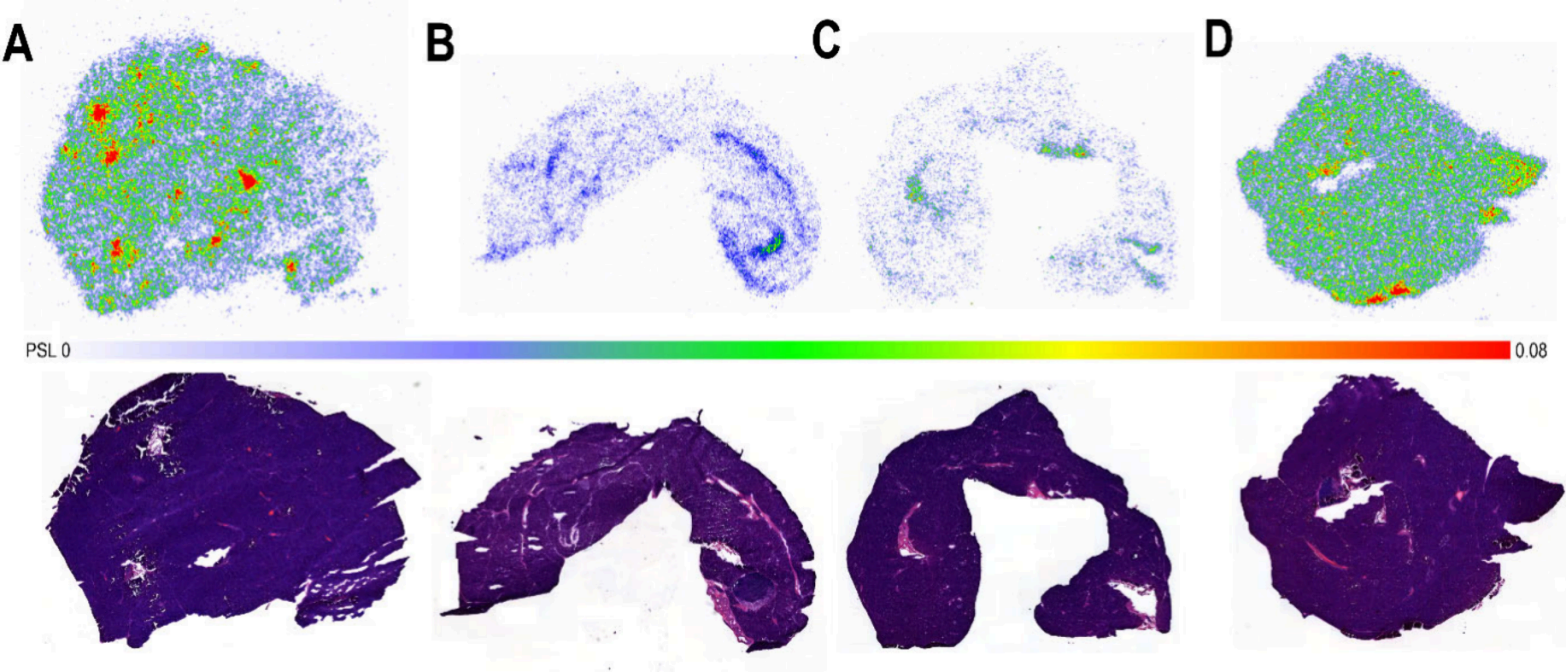
Representative *ex vivo* autoradiography images of the pancreas at 120 min after tracer injection. (A) Radioactivity distribution in pancreas after i.v. injection of [^18^F]­F-Tz-TCO-scFv 1F4 ([^18^F]**4**) (top image) and H&E heart (bottom image), (B) after blocking with scFv 1F4 (**1**) (50 μg, 60 min prior to the tracer) (top image) and H&E heart (bottom image), (C) after injection of [^18^F]­F-Tz-TCO-scFv 14b7* ([^18^F]**5**) (top image) and H&E heart (bottom image), and (D) radioactivity distribution after the pretargeted approach (10 μg TCO-scFv 1F4 (**4**) 60 min prior to [^18^F]­AmBF_3_-Tz ([^18^F]**3**); pancreas harvested 60 min after the tracer injection) (top image) and H&E heart (bottom image). Photostimulated luminescence (PSL); maximum and minimum values indicated.

## Discussion and Conclusions

The aim of this study was to develop a radiolabeled antibody fragment tracer targeting the α1 subunit of the GABA-A receptor in both humans and rodents, enabling investigation of the ion channel distribution in the nervous system using PET. To achieve high specificity, an immunoPET tracer, [^18^F]**4**, was designed instead of a low-molecular-weight compound. To our knowledge, radiolabeled antibody fragment [^18^F]**4** is the first PET tracer specifically targeting the α1 subunit of the GABA-A receptor, and this study represents the first application of immunoPET to explore the GABA-A receptor distribution.

Most PET-compatible positron emitting radionuclides have short physical half-lives (*t*_1/2_ ranging from minutes to hours), which impose a strict pharmacokinetic requirement on immunoPET tracers. For optimizing pharmacokinetics without compromising specificity of binding, we decided to engineer a scFv tracer from the anti-α1 subunit of GABA-A receptor mAb 1F4, which was selected based on GABA-A receptor interaction analysis of its cryo-EM structure. Encouraged by the *in silico* results indicating that mAb 1F4 binds specifically to the mouse GABA-A α1 subunit, antibody fragment **1** was designed and produced in Expi293f cells. The recombinant fragment was isolated with a *M*_w_ of 28.39 kDa, a size better matched to the short half-life of fluorine-18 (^18^F; *t*_1/2_ = 109.7 min) than that of the full-length mAb. Potential pharmacological effects of mAb 1F4 and **1** were evaluated electrophysiologically on cells expressing α1 subunit-containing GABA-A receptors. At the concentration of 1 μM, no effects on GABA-activated chloride currents were observed. Previous studies have shown similar results with anti-α1 subunit scFvs targeting a 15-amino acid N-terminal peptide.^29^ Interestingly, autoimmune encephalitis from anti-GABA-A antibodies is rare (3% of cases), and these antibodies block either the neurotransmitter or benzodiazepine binding sites.^30^ The structural data on mAb 1F4 confirm that it does not bind these sites.^31,32^ This, together with the fact that PET-tracers are typically injected at subpharmacological trace concentrations, further reduces the risk of unwanted pharmaceutical effects. Antibody fragment **1** was functionalized with TCO and efficiently ^18^F-labeled under mild reaction conditions in high RCY by biorthogonal inverse electron demand Dield-Alder (iEDDA) ligation between the TCO-conjugated scFv and radiolabeled compound [^18^F]**3**.

PET evaluation of [^18^F]**4** biodistribution in mice revealed favorable pharmacokinetics and the α1 subunit of GABA-A receptor specific binding in multiple GABA-A receptor-expressing organs, including the brain, heart, and lungs. The specific binding to the GABA-A receptor α1 subunit was confirmed by successful blocking with antibody fragment **1** and by comparison to a negative control fragment [^18^F]**5**. Binding specificity of [^18^F]**4** was further investigated by *ex vivo* biodistribution, autoradiography, and heart immunofluorescence staining. Notably, binding was observed in blood cells, including immune cells such as macrophages and T cells, which are reported to express functional GABA-A receptors. Accordingly, the cardiac PET signal likely reflects a combination of neuronal, cardiac, and immune cell receptor expression rather than the neuronal α1 subunit alone. Autoradiography demonstrated a strong, specific signal in the atrial region of the heart, where cardiac neurons reside along with other areas. Immunofluorescence with the full-size mAb 1F4 corroborated these findings, confirming **1** specificity through blocking experiments. Furthermore, high specific binding of [^18^F]**4** was observed in the lungs. The expression of ionotropic GABA receptors may be regulated in the lungs due to the critical role of Cl^–^ transport in lung development. In fact, it has been reported that the α1 subunit of GABA-A receptors is expressed on the apical membranes of adult lung alveolar type II cells in rats, and this expression increases from day 18 of gestation to the adult stage.^33–35^

Passage of [^18^F]**4** across the blood-brain barrier (BBB) was low as expected, but *ex vivo* autoradiography of the brain revealed a weak but specific signal, confirming the specificity of the faint brain accumulation, which was quantified based on PET and *ex vivo* biodistribution studies. Interestingly, brain areas with the most apparent signal were the cortex and cerebellum, matching the areas with the most prominent α1 subunit expression reported in the brain.^36^

GABAergic signaling is involved in various neurons and their innervations in the gut and endocrine organs, where it stimulates motor neurons and non-neural cells via GABA-A receptors.^37,38^ Consistent with this, the *ex vivo* biodistribution of [^18^F]**4** showed specific binding in the spleen, pancreas, Harderian glands, liver, and large intestine. Most interestingly, the *ex vivo* autoradiographic analysis of the pancreas revealed a dotted binding pattern, which was blocked by antibody fragment **1**. The functional GABA-A receptors are expressed in both α-cells and β-cells,^39^ and a recent study with [^11^C]­flumazenil suggested that GABA-A receptor expression in β-cells could serve as a potential marker for quantifying endocrine cell destruction in type 1 diabetes (T1D). However, while [^11^C]­flumazenil was bound to GABA-A receptors in pancreatic islets in the guinea pig, the contrast and signal strength were found to be insufficient for implementation as an *in vivo* PET marker for measuring pancreatic mass.^40^ The presence of the α1 subunit has been described in rat pancreatic tissue as well as in human islets, but in mice, the results have been contradictory.^41–44^ However, two studies have identified specific α1 subunit expression in β-cells in C57BL/6J mice and Kunming strains using immunohistochemistry.^45,46^ Further experiments are required to corroborate our preliminary finding that [^18^F]**4** binds the α1 subunit of the GABA-A receptor in the pancreas and the potential of the α1 subunit of GABA-A receptor specific PET tracers to measure changes in pancreatic endocrine cell mass.

PET evaluation of [^18^F]**4** binding to α1 subunit of GABA-A receptor by using the tracer with high and low *A*_m_ revealed competition for binding with increasing carrier amount and highlighted the importance of using a tracer with high *A*_m_. The tracer with high *A*_m_ resulted in significantly greater uptake in the heart, lungs, and brain compared to the tracer with low *A*_m_. *Ex vivo* analysis revealed further competition in blood, plasma, blood cells, spleen, pancreas, bladder, and Harderian glands when the tracer with low *A*_m_ was used. The gallbladder uniquely exhibited increased uptake, analogous to the blocked conditions.

Pretargeted PET imaging of the α1 subunit of GABA-A receptor was also investigated, but it failed to improve target to background ratios. The pretargeted approach revealed increased uptake in several organs such in the heart, brain, lungs, and spleen, but the results were not corroborated *ex vivo*. Elevated blood and plasma radioactivity in the pretargeted group confirmed successful reaction of intravenously injected radiolabeled compound [^18^F]**3** with modified antibody fragment **4** bound to α1 subunit of GABA-A receptors in blood cells and circulating immune cells but with lower overall signal intensity than with [^18^F]**4**. Furthermore, autoradiographic analysis confirmed specific binding in the heart but similarly with lower signal intensity than with the targeted approach. Obviously, the engineered [^18^F]**4** exhibited favorable pharmacokinetics, and the pretargeted approach was not able to provide any further benefit, although the pretargeted approach has been successfully used by several research groups for improving target to background ratios with full sized antibodies.^47,48^

To our knowledge, radiolabeled antibody fragment [^18^F]**4** is the first immunoPET tracer developed for the α1 subunit of the GABA-A receptor. Biological evaluation of [^18^F]**4** in mice confirmed favorable pharmacokinetics and specific binding to the α1 subunit of GABA-A receptors. The work demonstrates the potential of immunoPET for targeting complex ion channels such as GABA-A receptors with high specificity. Radiolabeled antibody fragment [^18^F]**4** is a promising PET tracer for assessing the α1 subunit of GABA-A receptor distribution and expression levels in peripheral organs, especially in the heart. Future directions include developing antibody fragment formats capable of crossing the BBB and applying the approach in disease models.

## Experimental Section

### General Information

All chemicals and reagents were of analytical grade and used without further purification. General chemicals and consumables were sourced from Merck (including Sigma-Aldrich, Darmstadt, Germany). Potassium hydrogen fluoride (KHF_2_, 99%) was purchased from abcr GmbH (Karlsruhe, Germany) and pyridazine (>99%), from Tokyo Chemical Industry (TCI, Tokyo, Japan). Both tetrazine-NSH ester (95%) and *trans*-cyclooctene (TCO)-PEG_4_-NHS ester (95%) were from BroadPharm (San Diego, CA, USA). Sep-Pak C18 Plus Short ( 360 mg sorbent per cartridge, 55–105 μm) and Sek-Pak C18 Plus Long (360 mg sorbent per cartridge, 55–105 μm) cartridges were supplied from Waters Corporation (Millford, MA, USA). AG 1-X8 resin (200–400 mesh, hydroxide form, biotechnology grade) was acquired from Bio-Rad (Hercules, CA, USA), and PD MiniTrap G-25 columns were obtained from Cytiva (Marlborough, MA, USA). Ultrapure water (18.2 MΩ·cm at 25 °C) was used in all experiments, sourced from a Milli-Q purification system (Merck Millipore, MA, USA) or as TraceSELECT water (Honeywell, NJ, USA). All solutions used for antibody fragment conjugation, radiolabeling, and analysis were sterile-filtered through a 0.22 μm membrane. Nuclear magnetic resonance (NMR) spectra, including ^1^H, ^13^C, ^19^F, COSY, multiplicity edited HSQC (CH and CH_3_ positive, CH_2_ negative, both coupled and decoupled), and HMBC, were recorded on a Bruker Avance-III spectrometer equipped with a Smartprobe BB/1H operating at 500.06 MHz (^1^H), 125.75 MHz (^13^C), and 470.48 MHz (^19^F). The measured NMR spectra were processed by using TopSpin software (version 3.6.4), and chemical shifts were referenced to residual solvent signals. For quadrupole time-of-flight mass spectrometry (Q-TOF-MS), a TripleTOF 6600 mass spectrometer was used (Sciex, Framingham, MA, USA). Thin-layer chromatography (TLC) was performed on silical gel 60 F_254_ or RP-18 F_254S_ plates (Merck) using methanol/chloroform (10:90, v/v) or water/acetonitrile (20:80, v/v) as the mobile phase, respectively. Nonradioactive compounds were visualized under UV light. For radiochemical analysis, phosphor storage screens (BAS-TR2025; Fujifilm) were exposed to developed TLC plates and scanned using a BAS-1800 II system (Fujifilm, Tokyo, Japan). Images were processed using Tina software (version 2.10f; Elysia-raytest GmbH, Straubenhardt, Germany). Radio-HPLC and radio-SEC-HPLC were performed using a Shimadzu Nexera 40 HPLC system (Kyoto, Japan), equipped with a SPD-40 UV–vis detector, operated via *LabSolutions* software. A NaI­(Tl) scintillation detector (Ø 2 × 2 in.) was connected in series with a UV detector for radioactivity detection. Radio-HPLC was conducted using a Jupiter Proteo C18 column (4 μm, 90 Å, 250 × 4.6 mm; Phenomenex) with isocratic elution of 25% solvent B (solvent A, water with 0.1% trifluoroacetic acid (TFA) in water; solvent B, acetonitrile with TFA) at flow rate of 2 mL/min and monitored at 254 nm. Radio-SEC-HPLC was carried out on a Superdex 75 increase 10/300 GL column (Cytiva, Marlborough, MA, USA) with PBS (0.01 M, pH 7.4) as the mobile phase at flow rate of 0.3 mL/min. The detection wavelength was set at 214 and 280 nm. Protein concentrations were measured using a NanoDrop OneC Microvolume UV–vis spectrophotometer (Thermo Fisher Scientific, Waltham, MA, USA). All tested compounds were ≥95% pure as determined by analytical HPLC.

#### Synthesis and Characterization of AmBF_3_-Tz (**3**)

##### 2-[4-(1,2,4,5-Tetrazin-3-yl)­phenyl]-*N*-[2-(dimethylamino)­ethyl]­acetamide

Dry dichloromethane (2 mL) and *N*,*N*-dimethylethylene-1,2-diamine (52 μL, 0.48 mmol) were charged into two necked round-bottom flasks under nitrogen. 3-(4-Phenylacetic acid)-1,2,4,5-tetrazine succinimidyl ester (100 mg, 0.32 mmol) was dissolved into dry dichloromethane (20 mL) and added portion wise within 20 min. The mixture was stirred at RT for 2 h, followed by evaporation of solvent. The crude mixture was purified by silica gel flash column chromatography (isocratic: 10% MeOH/CHCl_3_) to afford 85 mg of red solid containing ∼12 wt % *N*-hydroxysuccinimide, which was used in the next step. Yield: 75 mg, 82%, *R*_f_ = 0.09 (10% MeOH/CHCl_3_). NMR data are consistent with previously published data.^27^ ^1^H NMR (500.06 MHz, CDCl_3_) δ 10.19 (s, 1H), 8.56 (d, *J* = 8.3 Hz, 2H), 7.55 (d, *J* = 8.3 Hz, 2H), 6.85 (b, 1H), 3.68 (s, 2H), 3.41 (q, *J* = 5.6 Hz, 2H), 2.56 (t, *J* = 5.6 Hz, 3H), 2.31 (s, 6H). ^13^C NMR (125.75 MHz, CDCl_3_) δ 170.4, 166.5, 157.9, 141.0, 130.5, 130.4, 128.7, 57.9, 44.8, 43.6, 36.6.

##### 2-(2-(4-(1,2,4,5-Tetrazin-3-yl)­phenyl)­acetamido)-*N*,*N*-dimethyl-*N*-((4,4,5,5-tetramethyl-1,3,2-dioxaborolan-2-yl)­methyl)­ethan-1-aminium iodide

Dry MeCN (2 mL) and 2-(iodomethyl)-4,4,5,5-tetramethyl-1,3,2-dioxaborolane (80 mg, 0.3 mmol) were charged into two necked round-bottom flasks under nitrogen. 2-[4-(1,2,4,5-tetrazin-3-yl)­Phenyl]-*N*-[2-(dimethylamino)­ethyl]­acetamide (85 mg, ∼88% pure, 0.26 mmol) was added to dry MeCN (4 mL), and the mixture was stirred for 19 h followed by evaporation of the solvent. Crude product was dissolved in acetonitrile (2 mL) and then slowly added dropwise to slowly stirred diethyl ether (30 mL). Precipitate was allowed to stir for 1 h after which the solvent was decanted. Obtained solid powder was triturated twice for 1 h with diethyl ether (20 mL each). Obtained pale red powder was evaporated to dryness (137 mg), containing ∼5 wt % of 2-[4-(1,2,4,5-tetrazin-3-yl)­phenyl]-*N*-[2-(dimethylamino)­ethyl]­acetamide, which was used in the next step. Yield: 130 mg, 90%. NMR data are consistent with previously published data.^27^ ^1^H NMR (500.06 MHz, CD_3_CN) δ 10.28 (s, 1H), 8.52 (d, *J* = 8.2 Hz, 2H), 7.57 (d, *J* = 8.2 Hz, 2H), 7.33 (b, 1H) 3.66 (s, 2H), 3.57 (q, *J* = 6.0 Hz, 2H), 3.44 (t, *J* = 6.0 Hz, 2H), 3.18 (s, 2H), 3.11 (s, 6H), 1.28 (s, 12H). ^13^C NMR (125.75 MHz, CD_3_CN) δ 171.8, 167.3, 159.0, 141.9, 131.6, 131.4, 129.1, 86.6, 66.0, 58.6, 54.7, 43.4, 34.7, 25.0.

##### {[(2-{2-[4-(1,2,4,5-Tetrazin-3-yl)­phenyl]­acetamido}­ethyl)-dimethylammonio]­methyl}-trifluoroborate (AmBF_3_-Tz)

2-(2-(4-(1,2,4,5-Tetrazin-3-yl)­phenyl)­acetamido)-*N*,*N*-dimethyl-*N*-((4,4,5,5-tetramethyl-1,3,2 -dioxaborolan-2-yl)­methyl)­ethan-1-aminium iodide (137 mg, 95%, 0.23 mmol) was dissolved into DMF (3 mL) in a 50 mL Falcon (LDPE) tube. To the mixture was added 3 M KHF_2_ (aq, 1.2 mL) and 4 M HCl (aq, 1.2 mL). The solution was heated to 70 °C and stirred for 30 min followed by cooling to RT. The mixture was diluted with H_2_O (30 mL) and divided into 2 Sep-Pak C18 Plus Long cartridges (820 mg of C18; Precondition: 8 mL of MeCN, 30 mL of H_2_O). The cartridges were washed with H_2_O (30 mL), dried under N_2_ stream, and then eluted with MeCN (8 mL). Combined organics were evaporated and purified by reverse phase flash chromatography (Gradient: 20–100% H_2_O/MeCN) yielding a red powder. Yield: 68 mg, 86%, *R*_f_ = 0.71 (C18-TLC 20:80 H_2_O:MeCN). NMR-data are consistent with previously published data.^27^ ^1^H NMR (500.06 MHz, CD_3_CN) δ 10.27 (s, 1H), 8.52 (d, *J* = 8.3 Hz, 2H), 7.55 (d, *J* = 8.3 Hz, 2H), 6.78 (b, 1H), 3.62 (s, 2H), 3.58 (q, *J* = 6.6 Hz, 2H), 3.32 (t, *J* = 6.6 Hz, 2H), 2.98 (s, 6H), 2.35 (b, 2H). ^13^C NMR (125.75 MHz, CD_3_CN) δ 171.5, 167.3, 159.0, 142.0, 131.8, 131.4, 129.1, 65.4, 61.3, 54.3, 43.4, 34.8, 30.3. ^19^F NMR (470.48 MHz, CD_3_CN) δ −138.8, −138.9, −139.0, −139.1.

### Identification and Assessment of the Anti-α1 Subunit of GABA-A Receptor Antibody

The sequence of mAb 1F4 against the human α1β2γ2 GABA-A receptor was obtained from the Protein Data Bank (PDB: 6D6T and 6X3Z). To verify that the antibody fragment 1F4 binds specifically to the α1 subunit of GABA-A receptor, protein interaction analysis was performed using Maestro BioLuminate (Schrödinger Inc., NY) for the PBD structures 6D6T and 6X3Z showing the complex of the antibody 1F4 against the human α1β2γ2 GABA-A receptor. Briefly, after preparing the proteins with Protein Preparation Workflow with default settings, the Protein Interaction Analysis tool was run for both antibody fragments separately (as Set1) against all subunits of the GABA-A receptor (as Set2) with default settings, except for the maximum distance of hydrogen bonds, which was set to 3.5 Å. The resulting interaction tables and diagrams were analyzed to identify residues with specific interactions. Next, a multiple sequence alignment of human α1 subunit and mouse α1 to α6 subunits of GABA-A receptor (UniProt P14867, P62812, P26048, P26049, Q9D6F4, Q8BHJ7, and P16305) was created using the online ClustalOmega tool. The interaction residues in the α1 subunit of the GABA-A receptor were analyzed in the context of multiple sequence alignment to assess the conservation and predict the likelihood of similar interactions with the α subunits of the GABA-A receptor.

### Production of Antibody Fragments scFv 1F4 (**1**) and scFv 14b7* (**2**)

mAb 1F4 was reformatted as a scFv with C-terminal C-myc and His-tags. As a negative control, the antibody fragment **2** was adapted to the scFv format too. The scFvs were cloned in the pTwist CMV vector (Twist Biosciences) for mammalian cell expression using *Hin*dIII and *Bam*HI sites.

Antibody fragments **1** and **2** were expressed by using the Expi293 Expression System Kit (Thermofisher). Briefly, Expi293f mammalian cells were transiently transfected with the expression vectors and cultured for 7 days. After this, **1** or **2** was then affinity purified in culture media using the HisPur Ni-NTA Resin (Thermofisher), followed by SEC with a Superdex 75 Increase 10/300 GL column. The samples were dialyzed with Slide-A-LyzerTM G3 Dialysis Cassettes (Thermofisher) in PBS (pH 7.4) and analyzed with polyacrylamide gel electrophoresis (SDS-PAGE). The yields of purified **1** and **2** were 23.3 ± 8.6 mg (*n* = 2) and 0.4 mg (*n* = 1), respectively.

### α1 Subunit-Containing WSS-1 Cell Electrophysiology

WSS-1 cells (ATCC CRL-2029), a human embryonic kidney (HEK) cell line stably expressing α1β3γ2 receptors,^49,50^ were maintained in Dulbecco’s modified Eagle’s medium supplemented with 10% fetal bovine serum (Gibco, Gaithersburg, MD; United States), 50 U/mL penicillin, and 50 μg/mL streptomycin (Sigma-Aldrich, St Louis, MO, United States) at 37 °C, 95% air under 5% CO_2_. Cells were passaged and plated on 12 mm coverslips and incubated for 48–72 h before whole-cell patch clamp recording.

Cells were clamped at −60 mV at RT (20:22 °C) using an Axopatch 200B amplifier (Molecular Devices, Sunnyvale, CA, USA). Data were digitized and analyzed with NI-DAQ (National Instruments, Austin, TX, USA) and the Strathclyde Electrophysiology Software Package WinWCP (University of Strathclyde, UK). Patch pipettes (3–5 MΩ) were pulled from borosilicate glass (1.5/1 12 mm; OD/ID; WPI, Sarasota, FL, USA) using a P-87 Flaming Brown micropipette puller (Sutter Instrument Company, Rafael, CA, USA) and filled with an intracellular solution containing (in mM): 150 CsCl, 2 MgCl_2_, 1.1 EGTA, 2 Mg-ATP, and 10 HEPES, pH 7.4, adjusted with 1 mM CsOH.

Cells were continuously perfused with external HEK-Krebs solution containing (in mM): 140 NaCl, 4.7 KCl, 1.2 MgCl_2_, 2.52 CaCl2, 11 glucose, and 5 HEPES, pH 7.4, adjusted with 1 mM NaOH.

GABA stock solutions were diluted to 10 μM, and antibodies (antibody fragment **1** or a commercial mAb 1F4, Abcam, and Ab281915) were diluted to 1 μM in external solutions. Solutions were gravity-perfused at 3–4 mL/min at RT. GABA was administered in the bath for 1 min, followed by antibody application for 3–4 min. The peak amplitudes were measured directly from baseline to peak response.

### Radiosynthesis of [^18^F]­AmBF_3_-Tz ([^18^F]**3**)

No-carrier-added [^18^F]­fluoride was produced via ^18^O­(p,n)^18^F nuclear reaction using a TR-19 cyclotron (Advanced Cyclotron Systems Inc., Richmond, Canada) by proton irradiation of ^18^O-enriched water (>97%, Rotem Industries, Israel). Semiautomated radiosynthesis was carried out on a custom, remote-controlled radiosynthesis module (DM-automation, Nykyarn, Sweden). An integrated system was developed by coupling an HPLC injector to a radiosynthesis module for controlled [^18^F]­fluoride trapping and elution. Additionally, a self-assembled anion-exchange cartridge (AEC) was prepared using AG 1-X8 resin (2.3–2.5 mg) packed into PTFE tubing. A pyridazine-HCl buffer (pH 2.0–2.2) was employed as both the eluent and the reaction buffer. The optimized buffer composition for our setup was as follows (v/v): pyridazine (19.57%), H_2_O (29.35%), 10 M HCl (15.22%), and acetonitrile (35.87%). The sample loop of the integrated HPLC injector served as a reservoir for the pyridazine-HCl buffer.

At the start of the radiosynthesis, the integrated HPLC injector was set to the load position, and aqueous [^18^F]­fluoride (6–13 GBq) was passed through the injector and directed through the custom AEC, where [^18^F]­fluoride ions were trapped while the target water was diverted into a waste vial. After trapping, nitrogen was blown through the AEC for 1 min to dry the resin bed. The injector was then switched to the inject position, and 10–15 μL of pyridazine-HCl buffer was pushed through the AEC into an Eppendorf vial preloaded with 150 nmol of the **3** precursor. The reaction mixture was heated at 85 °C for 10 min, diluted with 4 mL of Milli-Q water, and purified via semipreparative HPLC using a C18 Jupiter Proteo column (250 × 10 mm, 4 μm, 90 Å; Phenomenex); eluent A: water with 0.1% trifluoroacetic acid (TFA); eluent B: acetonitrile with 0.1% TFA; isocratic elution at 25% B; flow rate: 5 mL/min; R_t_ = 9 min. Purified [^18^F]**3** was collected into a glass vial containing 40 mL of Milli-Q water and trapped on a Sep-Pak C18 Plus Short cartridge. The cartridge was washed with 25 mL of water and flushed with N_2_. Radiolabeled compound [^18^F]**3** was eluted with 500 μL of 50% EtOH/H_2_O (v/v), followed by 1 mL of 0.01 M PBS into the product vial preloaded with 1 mL of PBS (0.01 M). The final product was analyzed by radio-HPLC and radio-TLC.

### TCO Modification of scFv 1F4 (**1**) and scFv 14b7* (**2**) and Characterization

An aliquot of **1** (283.9 μg, 10 nmol) was reacted with 15 equiv of TCO-PEG_4_-NHS ester (in DMSO) in a total volume of 1 mL of PBS (pH 8.5, adjusted with 0.1 M sodium carbonate). The reaction mixture was incubated at RT for 4 h in the dark. Subsequently, the reaction mixture was purified to remove unreacted TCO-PEG_4_-NHS ester using Amicon Ultra-0.5 centrifugal filter units with a 10 kDa molecular weight cutoff with PBS (pH 7.4, six washes, 10 min each, 14000*g*). The concentration of the purified conjugate was determined by UV absorbance at 280 nm using a NanoDrop spectrophotometer, and product integrity was confirmed by Q-TOF-MS based on the mass difference between the unconjugated and TCO-conjugated **1**. The antibody fragment **2** was modified by following the same protocol.

### *In Vitro* Radiolabeling of TCO-scFv 1F4 (**4**) and TCO-scFv 14b7*­(**5**)

TCO-functionalized antibody fragments **4** and **5** were radiolabeled via tetrazine (Tz) ligation with [^18^F]**3**. Briefly, freshly prepared and purified [^18^F]**3** (in PBS containing <10% ethanol, ∼480 μL) was added to **4** (3 nmol in ∼20 μL of PBS, pH 7.4) with Tz present in molar excess. The reaction mixture was incubated at RT for 10 min. The resulting radiolabeled construct, [^18^F]**4**, was purified using a PD MiniTrap G-25 column (Cytiva) pre-equilibrated with 0.01 M PBS, using PBS as the eluent, following the manufacturer’s instruction. Radiochemical purity was assessed by radio-TLC and radio-SEC-HPLC. Radiolabeled antibody fragment [^18^F]**5** was radiosynthesized using the same procedure, starting with 3 nmol of **5**.

### Animals

This study used female and male C57BL/6J mice (*n* = 50; 12 females; weight 27.49 ± 4.63 g). In C57BL/6J mice, CNS and PNS α1 subunit GABA-A receptor expression has been molecularly profiled.^2^ Animals were group-housed under standard laboratory conditions (21 ± 1.2 °C, humidity 55 ± 5%, with a 12-h light/dark cycle) with *ad libitum* access to tap water and soy-free chow (RM3 [E] soya-free, 801,710, Special Diets Service, UK). All procedures were approved by the Regional State Administrative Agency for Southern Finland (ESAVI/4499/04.10.07/2016) and complied with the European Union Directive 2010/EU/63 on the protection of animals used for scientific purposes.

### Small Animal PET/CT Imaging

Biodistribution of radiotracers *in vivo* was evaluated in C57BL/6J mice. All injections were performed intravenously via the tail vein using PBS. For targeted imaging, mice received [^18^F]**4** (3.71 ± 0.19 MBq, 3.79 ± 0.80 μg, *n* = 4). Target-binding specificity was evaluated by preinjecting 50 μg of unlabeled antibody fragment **1** 1 h before [^18^F]**4** (3.81 ± 0.05 MBq, 3.68 ± 0.54 μg, *n* = 4) administration, as well as by using the nontargeting control tracer [^18^F]**5** (3.33 ± 0.20 MBq, 2.31 ± 0.50 μg, *n* = 4). PET/CT imaging was performed on all groups with a 30 min static acquisition window, starting 90 min postradiotracer injection.

Furthermore, to evaluate the impact of the carrier competition on GABA-A receptor α1-subunit binding, mice were injected with either high or low *A*_m_ formulations of [^18^F]**4** (high: 3.76 ± 0.28 MBq, 6.62 ± 2.90 μg, *n* = 8; low: 2.57 ± 0.68 MBq, 12.08 ± 2.37 μg, *n* = 6). Imaging was conducted 210 min postinjection with a 30 min static acquisition.

For pretargeted PET imaging, mice received **4** (10 μg) followed, after 1 h, by [^18^F]**3** (3.18 ± 1.09 MBq). Mice were imaged either dynamically (timeframes 30 × 10 s, 15 × 60 s, 4 × 300 s, and 2 × 600 s) for 60 min immediately following [^18^F]**3** injection (*n* = 8) or statically for 30 min after a 90 min uptake period to allow *in vivo* biorthogonal ligation. Control groups received [^18^F]**3** alone (3.25 ± 1.11 MBq) and were imaged under identical conditions (*n* = 4 per group) to assess nonspecific uptake.

PET/CT imaging was performed using β-CUBE microPET and X-CUBE microCT scanners (MOLECUBES, Gent, Belgium). Mice were anesthetized with 2–2.5% isoflurane in 700 mL/min of oxygen, and scanning began immediately thereafter. Mice were imaged using CT for attenuation correction and anatomical reference, followed by PET imaging with an energy window of 511 keV ± 30%. Image data were reconstructed using the three-dimensional ordered-subset expectation maximization (OSEM3D) algorithm. Reconstructed DICOM images generated by the β-CUBE and X-CUBE scanners were converted using the PMOD software (version 4.0) into a format compatible with Inveon Research Workplace (v. 4.2).

Standardized volumes of interest (VOIs) were manually defined using the CT images for organs of interest (brain, heart, lungs, stomach, liver, spleen, pancreas, kidneys, muscle, and tibia as bone), except for the gallbladder, which was delineated using combined CT and PET information. Activity concentrations were corrected for decay and normalized to the injected dose, and the results are expressed as the mean percentage of injected dose per gram of tissue (%ID/g).

### *Ex Vivo* Biodistribution and Autoradiography

Immediately following PET/CT imaging, mice were euthanized under anesthesia via a terminal cardiac puncture. Blood was collected and centrifuged to separate the plasma and cells. Organs and tissues, including brain, heart, lung, kidneys, liver, spleen, pancreas, intestines (walls and contents), stomach, bladder with urine, gallbladder, Harderian glands, and gonads, were dissected, weighed, and assayed for radioactivity with a γ-counter (Wizard2 3″, PerkinElmer, Turku, Finland). The radioactive decay was corrected for the time of injection. Radioactivity uptake is expressed as ID%/g.

For autoradiography, samples of brain, heart, and pancreas were immediately frozen in isopentane cooled on dry ice and cut into 20 μm slices using a cryostat (Leica CM3050S, Germany). The cryosections were thaw-mounted onto glass slides (Superfrost Ultra Plus, Thermo Fisher, USA) and exposed to BAS-TR2025 phosphorimaging plates (Fujifilm, Tokyo, Japan) for approximately two half-lives of fluorine-18. Plates were scanned at a 25 μm resolution and 16-bit gradation using a BAS-5000 reader (Fujifilm, Tokyo, Japan). Digital autoradiographic images were analyzed with AIDA Image Analyzer 4.5 software (Raytest, Isotopenmessgeräte, Straubenhardt, Germany). The data were corrected for radioactive decay based on injection and exposure times and adjusted for the administered activity.

### Immunofluorescence in Heart Sections

Fresh-frozen sections of mouse heart tissue were cryosectioned at a 20 μm thickness using a cryostat (Leica CM3050S, Germany), thaw-mounted onto microscope slides (Superfrost Ultra Plus, Thermo Fisher, USA), and air-dried. Afterward, sections were fixed in 4% paraformaldehyde in PBS (0.01 M, pH 7.4) for 30 min at RT and washed twice with PBS for 5 min each. Permeabilization and blocking was performed by incubating sections for 1 h at RT in blocking buffer consisting of 2% bovine serum albumin (BSA), 2% Goat Serum, and 0.2% Triton X-100 in PBS. Slides were then incubated for 24 h at 4 °C with a chimeric rabbit mAb targeting the α1 subunit of GABA-A receptors (mAb 1F4, Abcam, Ab281915), diluted 1:100 in blocking buffer. Next day, the slides were washed three times with PBS containing 0.2% Triton X-100 (pH 7.4). Sections were subsequently incubated at RT for 1 h with Alexa Fluor 568-conjugated goat antirabbit IgG (H+L) secondary antibody (Invitrogen), diluted 1:1000 in blocking buffer. After secondary antibody incubation, the slides were washed three times with PBS containing 0.2% Triton X-100 (pH 7.4). Nuclei were counterstained; sections were mounted using VECTASHIELD Hardset Antifade Mounting Medium with DAPI (Vector Laboratories), and coverslips were applied. For the control experiment, sections were preincubated with **1** (diluted 1:100 in blocking buffer) for 24 h prior to application of mAb 1F4, followed by secondary staining as described above.

Images of the stained slides were taken with a Panoramic Midi fluorescence slide scanner (3DHISTECH) and visualized with CaseViewer software (3DHISTECH).

### Statistical Analysis

The results are reported as average ± SD when *n* was at least 2. All statistical analyses were calculated using Prism programs (version 10.2.0; GraphPad Software). Differences in GABA-evoked currents were analyzed using the nonparametric paired sample Wilcoxon test. Differences in PET and biodistribution study groups were analyzed using the nonparametric multiple *t* test because of the small sample size. Differences were considered statistically significant if the *P* value was less than 0.05.

## Supplementary Material





## Notes

Additional tables and figures on the protein interaction analysis in 6D6T and 6X3Z structures from PDB, protein concentration measurement by UV absorbance, antibody fragment production, GABA-evoked current in WSS-1 cells, quality control of final purified [^18^F]­AmBF_3_-Tz ([^18^F]**3**), Q-TOF mass spectrometry analysis of scFv 1F4 (**1**), quality control of TCO-scFv 14b7* (**5**) and [^18^F]­F-Tz-TCO-scFv 14b7* ([^18^F]**5**), *ex vivo* biodistribution of [^18^F]­F-Tz-TCO-scFv 1F4 ([^18^F]**4**) (baseline and blocked), and [^18^F]**5**, *ex vivo* biodistribution of [^18^F]**4** after i.v. injection of the tracer with high or low molar activity (*A*_m_), *ex vivo* biodistribution after pretargeting α1 subunit of GABA-A receptors with (**4**) (10 μg i.v.) 60 min prior to [^18^F]**3**, and NMR-spectra of AmBF_3_-Tz (**3**) (PDF)
Molecular formula String file of compounds (CSV)

## Notes

Turku PET Centre, University of Turku and Turku University Hospital, 20520 Turku, Finland

## Abbreviations

*A*_m_: molar activity
ANS: autonomic nervous system
CNS: central nervous system
Cryo-EM: cryogenic electron microscopy
CT: computerized tomography
EOS: end of the synthesis
GABA: gamma-aminobutyric acid
HPLC: high performance liquid chromatography
IEX: isotope exchange
immunoPET: immune-positron emission tomography
kDa: kilodalton
NMR: nuclear magnetic resonance
mAb: monoclonal antibody
Max.: maximum
Min.: Minimum
OSEM3D: three-dimensional ordered-subsets expectation maximization algorithm
PET: positron emission tomography
PNS: peripheral nervous system
POH: pressure-overload hypertrophy
PSL: photostimulated luminescence
QC: quality control
Q-TOF-MS: quadrupole time-of-flight mass spectrometry
RCY: radiochemical yield
RT: room temperature
scFv: single-chain antibody variable fragment
SDS-PAGE: sodium dodecyl sulfate polyacrylamide gel electrophoresis
SEC: size exclusion chromatography
SOS: start of the synthesis
TCO: *trans*-cyclooctene
TLC: thin-layer chromatography
Tz: tetrazine
VOIs: volumes of interest

## References

[1] Qian X., Zhao X., Yu L., Yin Y., Zhang X.-D., Wang L., Li J.-X., Zhu Q., Luo J.-L. (2023). Current status of GABA receptor subtypes in analgesia. Biomed Pharmacother..

[2] Everington E. A., Gibbard A. G., Swinny J. D., Seifi M. (2018). Molecular
Characterization
of GABA-A Receptor Subunit Diversity within Major Peripheral Organs
and Their Plasticity in Response to Early Life Psychosocial Stress. Frontiers in Molecular Neuroscience.

[3] Ghit A., Assal D., Al-Shami A. S., Hussein D. E. E. (2021). GABA­(A) receptors:
structure, function, pharmacology, and related disorders. J. Genet Eng. Biotechnol..

[4] Crestani, F. ; Rudolph, U. Chapter Two - Behavioral Functions of GABAA Receptor Subtypes - The Zurich Experience. In AAdvances in Pharmacology; Rudolph, U , Ed.; Academic Press, 2015; Vol. 72, p 37–51. 10.1016/bs.apha.2014.10.001 25600366

[5] Engin E., Benham R. S., Rudolph U. (2018). An Emerging Circuit Pharmacology
of GABA­(A) Receptors. Trends Pharmacol. Sci..

[6] Rudolph U., Knoflach F. (2011). Beyond classical benzodiazepines: novel therapeutic
potential of GABAA receptor subtypes. Nat. Rev.
Drug Discovery.

[7] Akinci M. K., Schofield P. R. (1999). Widespread expression of GABA­(A) receptor subunits
in peripheral tissues. Neurosci Res..

[8] Poulter M. O., Singhal R., Brown L. A., Krantis A. (1999). GABA­(A) receptor subunit
messenger RNA expression in the enteric nervous system of the rat:
implications for functional diversity of enteric GABA­(A) receptors. Neuroscience..

[9] Bhat R., Axtell R., Mitra A., Miranda M., Lock C., Tsien R. W. (2010). Inhibitory
role for GABA in autoimmune inflammation. Proc.
Natl. Acad. Sci. U. S. A..

[10] Reyes-García M.
G., Hernández-Hernández F., Hernández-Téllez B., García-Tamayo F. (2007). GABA (A) receptor
subunits RNA expression in mice peritoneal
macrophages modulate their IL-6/IL-12 production. J. Neuroimmunol..

[11] Tian J., Lu Y., Zhang H., Chau C. H., Dang H. N., Kaufman D. L. (2004). Gamma-aminobutyric
acid inhibits T cell autoimmunity and the development of inflammatory
responses in a mouse type 1 diabetes model. J. Immunol..

[12] Hagan D. W., Ferreira S. M., Santos G. J., Phelps E. A. (2022). The role
of GABA
in islet function. Front Endocrinol (Lausanne).

[13] Rudolph U., Möhler H. (2014). GABAA receptor
subtypes: Therapeutic potential in Down
syndrome, affective disorders, schizophrenia, and autism. Annual review of pharmacology and toxicology.

[14] Egerton A., Modinos G., Ferrera D., McGuire P. (2017). Neuroimaging studies
of GABA in schizophrenia: a systematic review with meta-analysis. Translational psychiatry.

[15] Bryson A., Reid C., Petrou S. (2023). Fundamental
Neurochemistry Review:
GABA. J. Neurochem..

[16] Shi Y., Li Y., Yin J., Hu H., Xue M., Li X. (2019). A novel sympathetic
neuronal GABAergic signalling system regulates
NE release to prevent ventricular arrhythmias after acute myocardial
infarction. Acta Physiol (Oxf).

[17] Bu J., Huang S., Wang J., Xia T., Liu H., You Y. (2021). The GABA­(A) Receptor
Influences Pressure Overload-Induced
Heart Failure by Modulating Macrophages in Mice. Front Immunol..

[18] Andersson J. D., Halldin C. (2013). PET radioligands targeting the brain
GABAA /benzodiazepine
receptor complex. J. Labelled Comp Radiopharm..

[19] Lin S. F., Bois F., Holden D., Nabulsi N., Pracitto R., Gao H. (2017). The Search for a Subtype-Selective PET Imaging Agent
for the GABA. Mol. Imaging..

[20] de
Jonge J. C., Vinkers C. H., Hulshoff Pol H. E., Marsman A. (2017). GABAergic Mechanisms in Schizophrenia: Linking Postmortem
and In Vivo Studies. Frontiers in psychiatry.

[21] Murrell E., Pham J. M., Sowa A. R. (2020). Classics in Neuroimaging:
Development of Positron Emission Tomography Tracers for Imaging the
GABAergic Pathway. ACS Chem. Neurosci..

[22] de
Lucas Á G, Lamminmäki U., López-Picón F. R. (2023). ImmunoPET
Directed to the Brain: A New Tool for Preclinical and Clinical Neuroscience. Biomolecules.

[23] Wu A. M., Olafsen T. (2008). Antibodies for Molecular Imaging of Cancer. Cancer Journal.

[24] Fang X. T., Hultqvist G., Meier S. R., Antoni G., Sehlin D., Syvanen S. (2019). High detection
sensitivity with antibody-based PET
radioligand for amyloid beta in brain. Neuroimage.

[25] Zhu S., Noviello C. M., Teng J. (2018). Structure of a human
synaptic GABAA receptor. Nature.

[26] Kim J. J., Gharpure A., Teng J. (2020). Shared structural mechanisms
of general anaesthetics and benzodiazepines. Nature.

[27] Otaru S., Paulus A., Imlimthan S. (2022). Development of [18F]­AmBF3
Tetrazine for Radiolabeling of Peptides: Preclinical Evaluation and
PET Imaging of [18F]­AmBF3-PEG7-Tyr3-Octreotide in an AR42J Pancreatic
Carcinoma Model. Bioconjug Chem..

[28] Kwon D., Lozada J., Zhang Z. (2021). High-Contrast CXCR4-Targeted
18F-PET Imaging Using a Potent and Selective Antagonist. Mol. Pharmaceutics.

[29] Koduvayur S. P., Gussin H. A., Parthasarathy R., Hao Z., Kay B. K., Pepperberg D. R. (2014). Generation of Recombinant Antibodies
to Rat GABAA Receptor
Subunits by Affinity Selection on Synthetic Peptides. PLoS One.

[30] Kirschstein T., Köhling R. (2023). Functional changes in neuronal circuits
due to antibody-driven
autoimmune response. Neurobiol Dis..

[31] Tan K. R., Baur R., Charon S., Goeldner M., Sigel E. (2009). Relative positioning
of diazepam in the benzodiazepine-binding-pocket of GABA receptors. J. Neurochem..

[32] Newell J. G., Czajkowski C. (2003). The GABAA
receptor alpha 1 subunit Pro174-Asp191 segment
is involved in GABA binding and channel gating. J. Biol. Chem..

[33] Jin N., Kolliputi N., Gou D., Weng T., Liu L. (2006). A novel function
of ionotropic gamma-aminobutyric acid receptors involving alveolar
fluid homeostasis. J. Biol. Chem..

[34] Jin N., Guo Y., Sun P. (2008). Ionotropic GABA receptor expression in the
lung during development. Gene Expr Patterns.

[35] Chintagari N. R., Jin N., Gao L., Wang Y., Xi D., Liu L. (2010). Role of GABA
receptors in fetal lung development in rats. PLoS One.

[36] Hörtnagl H., Tasan R. O., Wieselthaler A., Kirchmair E., Sieghart W., Sperk G. (2013). Patterns of mRNA and
protein expression
for 12 GABAA receptor subunits in the mouse brain. Neuroscience.

[37] Poulter M. O., Singhal R., Brown L. A., Krantis A. (1999). GABA­(A) receptor subunit
messenger RNA expression in the enteric nervous system of the rat:
implications for functional diversity of enteric GABA­(A) receptors. Neuroscience.

[38] Li Y., Xiang Y. Y., Lu W. Y., Liu C., Li J. (2012). A novel role
of intestine epithelial GABAergic signaling in regulating intestinal
fluid secretion. Am. J. Physiol Gastrointest
Liver Physiol..

[39] Bansal P., Wang Q. (2008). Insulin as a physiological
modulator of glucagon secretion. Am. J. Physiol
Endocrinol Metab..

[40] Maloum-Rami F., Cheung P., Antoni G., Jin Z., Eriksson O., Espes D. (2024). PET imaging of GABAA receptors in
pancreatic islets by [11C]­flumazenil. EJNMMI
Res..

[41] Wendt A., Birnir B., Buschard K. (2004). Glucose inhibition of
glucagon secretion from rat alpha-cells is mediated by GABA released
from neighboring beta-cells. Diabetes.

[42] Borboni P., Porzio O., Fusco A., Sesti G., Lauro R., Marlier L. N. (1994). Molecular and cellular characterization of the GABAA
receptor in the rat pancreas. Mol. Cell. Endocrinol..

[43] von
Blankenfeld G., Turner J., Ahnert-Hilger G. (1995). Expression of functional GABAA receptors in neuroendocrine gastropancreatic
cells. Pflugers Arch..

[44] Xu E., Kumar M., Zhang Y. (2006). Intra-islet insulin
suppresses glucagon release via GABA-GABAA receptor system. Cell Metab..

[45] Feng A. L., Xiang Y. Y., Gui L., Kaltsidis G., Feng Q., Lu W. Y. (2017). Paracrine GABA and insulin regulate
pancreatic alpha cell proliferation in a mouse model of type 1 diabetes. Diabetologia.

[46] Wang S., Luo Y., Feng A. (2014). Ethanol induced impairment of glucose metabolism
involves alterations of GABAergic signaling in pancreatic β-cells. Toxicology.

[47] Zeglis B. M., Sevak K. K., Reiner T. (2013). A pretargeted
PET imaging
strategy based on bioorthogonal Diels-Alder click chemistry. J. Nucl. Med..

[48] Lumen D., Vugts D., Chomet M. (2022). Pretargeted PET Imaging
with a TCO-Conjugated Anti-CD44v6 Chimeric mAb U36 and [^89^Zr]­Zr-DFO-PEG_5_-Tz. Bioconjug Chem..

[49] Wong G., Sei Y., Skolnick P. (1992). Stable expression of type I γ-aminobutyric acidA/benzodiazepine
receptors in a transfected cell line. Mol. Pharmacol..

[50] Davies P. A., Hoffmann E. B., Carlisle H. J., Tyndale R. F., Hales T. G. (2000). The influence
of an endogenous β3 subunit on recombinant GABAA receptor assembly
and pharmacology in WSS-1 cells and transiently transfected HEK-293
cells. Neuropharmacology.

