# Supplementary material for: Fluorine-18 Radiolabeled Single-Chain Antibody Variable Fragment 1F4 Targets α1-Subunit Gamma-Aminobutyric Acid Type A Receptors in Mice
Source: J Med Chem. 2026 Feb 9;69(4):4288–302. doi: 10.1021/acs.jmedchem.5c02984 (PMC12951459; doi:10.1021/acs.jmedchem.5c02984)
Supplement: Supplementary file 1 [file jm5c02984_si_001.pdf]

SUPPORTING INFORMATION:

Fluorine-18 Radiolabeled Single-Chain Antibody Variable Fragment 1F4 Targets α1-Subunit Gamma-Aminobutyric Acid Type A Receptors in Mice

Ángel García de Lucas[1,2,3], Negar A. Samani[1,4], Olli Moisio[1,4], Luciana Kovacs[1,4,5], Risto Savela[1,4], Sanna L. Soini[6], Sami Oksanen[3], Jatta S. Helin[1,2], Johan Rajander[7], Anu J. Airaksinen[1,4*], Urpo Lamminmäki[3] and Francisco López-Picón[1,2*]*

#Corresponding author

Ángel García de Lucas: ángel.garciadelucas@utu.fi

Anu Airaksinen: anu.airaksinen@utu.fi

Francisco López-Picón: francisco.lopez@utu.fi

1. Turku PET Centre, University of Turku and Turku University Hospital, FI-20520 Turku, Finland.

2. PET Preclinical Imaging Laboratory, Turku PET Centre, University of Turku, FI20520 Turku, Finland.

3. Department of Life Technologies, University of Turku, FI-20520 Turku, Finland.

4. Department of Chemistry, University of Turku, FI-20500 Turku, Finland.

5. VARHA, The Wellbeing Services County of Southwest Finland, FI-20520 Turku, Finland.

6. Integrative Physiology and Pharmacology, Institute of Biomedicine, University of Turku, FI-20520 Turku, Finland.

7. Accelerator Laboratory, Åbo Akademi University, FI-20520 Turku, Finland.

Contents

Table S1. Protein interaction analysis in 6D6T and 6X3Z structures from PDB S3

Protein Concentration measurement by UV absorbance S4

Figure S1. Antibody fragments production S5

Figure S2. GABA-evoked current in WSS-1 cells S6

Figure S3. Quality control of final purified [^{18}F]AmBF^{3}-Tz ([^{18}F]**3**) S7

Figure S4. Q-TOF mass spectrometry analysis of scFv 1F4 (**1**) S8

Figure S5. Quality control of TCO-scFv 14b7* (**5**) and [^{18}F]F-Tz-TCO-scFv 14b7* ([^{18}F]**5**) S9

Table S2. *Ex vivo* biodistribution of [^{18}F]F-Tz-TCO-scFv 1F4 ([^{18}F]**4**) (baseline and blocked), and [^{18}F]**5** S10

Table S3. *Ex vivo* biodistribution of [^{18}F]**4** after i.v. injection of the tracer with high (injected dose 6.62 ± 2.90 µg, n = 8) or low molar activity (A_m) (injected dose 12.08 ± 2.37 µg, n = 6) S11

Table S4. *Ex vivo* biodistribution after pretargeting α1 subunit of GABA-A receptors with TCO-scFv 1F4 (**4**) (10 µg i.v.) 60 min prior to [^{18}F]**3** S12

NMR-spectra of AmBF3-Tz (**3**) S13-S20

Structure	scFv	Set1 Residues	Set2 Residues	Distance(A)	Specific Interactions	Surface Complementarity	Set1 Buried SASA	Set2 Buried SASA
6X3Z	1F4 (I/J)	HC:ASP 31	GBRA1:LYS 42	2.1 A	1x hb to GBRA1:LYS 42	0.52	67.9%	73.2%
6X3Z	1F4 (I/J)	HC:ASP 31	GBRA1:LYS 71	2.6 A	1x hb to GBRA1:LYS 71	0.79	67.9%	53.9%
6X3Z	1F4 (I/J)	HC:ARG 102	GBRA1:GLU 170	2.3 A	1x hb to GBRA1:GLU 170	0.67	87.1%	93.3%
6X3Z	1F4 (I/J)	HC:TRP 103	GBRA1:TRP 171	2.3 A	1x hb to GBRA1:TRP 171	0.77	98.3%	100.0%
6X3Z	1F4 (I/J)	HC:ARG 50	GBRA1:GLU 174	2.6 A	1x hb to GBRA1:GLU 174	0.81	94.3%	100.0%
6X3Z	1F4 (I/J)	HC:TYR 35	GBRA1:GLU 174	2.4 A	1x hb to GBRA1:GLU 174	0.71	83.4%	100.0%
6X3Z	1F4 (I/J)	LC:TYR 92	GBRA1:ALA 176	1.9 A	1x hb to GBRA1:ALA 176	0.85	86.9%	58.4%
6X3Z	1F4 (I/J)	LC:TYR 92	GBRA1:VAL 198	2.2 A	1x hb to GBRA1:VAL 198	0.85	86.9%	95.5%
6X3Z	1F4 (I/J)	LC:TYR 28	GBRA1:VAL 198	2.4 A	1x hb to GBRA1:VAL 198	0.82	48.1%	95.5%
6X3Z	1F4 (I/J)	LC:GLY 30	GBRA1:SER 200	3.4 A	1x hb to GBRA1:SER 200	0.72	79.8%	99.4%
6X3Z	1F4 (L/K)	HC:ASP 31	GBRA1:LYS 42	2.2 A	1x hb to GBRA1:LYS 42	0.64	65.9%	69.5%
6X3Z	1F4 (L/K)	HC:ASP 31	GBRA1:LYS 71	2.2 A	, 1x salt bridge to GBRA1:L	0.78	65.9%	56.7%
6X3Z	1F4 (L/K)	HC:TRP 103	GBRA1:GLU 170	2.2 A	1x hb to GBRA1:GLU 170	0.79	99.6%	98.6%
6X3Z	1F4 (L/K)	HC:LYS 99	GBRA1:GLU 170	2.5 A	salt bridge to GBRA1:GLU	0.73	97.9%	98.6%
6X3Z	1F4 (L/K)	HC:TRP 103	GBRA1:TRP 171	2.3 A	1x hb to GBRA1:TRP 171	0.77	99.6%	100.0%
6X3Z	1F4 (L/K)	HC:ARG 50	GBRA1:GLU 174	2.2 A	2x hb to GBRA1:GLU 174	0.77	100.0%	100.0%
6X3Z	1F4 (L/K)	HC:TYR 35	GBRA1:GLU 174	3.1 A	1x hb to GBRA1:GLU 174	0.59	100.0%	100.0%
6X3Z	1F4 (L/K)	LC:TYR 92	GBRA1:ALA 176	1.9 A	1x hb to GBRA1:ALA 176	0.83	85.5%	60.3%
6X3Z	1F4 (L/K)	LC:TYR 92	GBRA1:VAL 198	2.2 A	1x hb to GBRA1:VAL 198	0.86	85.5%	95.9%
6X3Z	1F4 (L/K)	LC:TYR 28	GBRA1:VAL 198	2.3 A	1x hb to GBRA1:VAL 198	0.84	47.5%	95.9%
6X3Z	1F4 (L/K)	LC:THR 31	GBRA1:ASP 199	2.0 A	1x hb to GBRA1:ASP 199	0.79	56.6%	62.5%
6D6T	1F4 (I/J)	HC:ASP 31	GBRA1:LYS 71	3.1 A	1x hb to GBRA1:LYS 71	0.49	58.4%	39.9%
6D6T	1F4 (I/J)	HC:ARG 102	GBRA1:GLU 170	2.4 A	1x hb to GBRA1:GLU 170	0.44	84.3%	98.0%
6D6T	1F4 (I/J)	HC:TYR 33	GBRA1:THR 172	1.9 A	1x hb to GBRA1:THR 172	0.78	66.1%	32.8%
6D6T	1F4 (I/J)	HC:ARG 50	GBRA1:GLU 174	2.4 A	salt bridge to GBRA1:GLU	0.54	94.6%	98.5%
6D6T	1F4 (I/J)	LC:TYR 92	GBRA1:ALA 176	2.1 A	1x hb to GBRA1:ALA 176	0.85	84.6%	55.2%
6D6T	1F4 (I/J)	LC:TYR 92	GBRA1:VAL 198	2.5 A	1x hb to GBRA1:VAL 198	0.84	84.6%	91.4%
6D6T	1F4 (I/J)	LC:TYR 28	GBRA1:VAL 198	3.1 A	1x hb to GBRA1:VAL 198	0.82	37.3%	91.4%
6D6T	1F4 (I/J)	LC:THR 31	GBRA1:ASP 199	2.2 A	1x hb to GBRA1:ASP 199	0.42	46.9%	58.9%
6D6T	1F4 (I/J)	LC:TYR 32	GBRA1:SER 200	1.8 A	1x hb to GBRA1:SER 200	0.63	100.0%	94.6%
6D6T	1F4 (L/K)	HC:ASP 31	GBRA1:LYS 42	2.2 A	1x hb to GBRA1:LYS 42	0.42	59.4%	56.8%
6D6T	1F4 (L/K)	HC:ASP 31	GBRA1:LYS 71	3.4 A	1x hb to GBRA1:LYS 71	0.54	59.4%	30.4%
6D6T	1F4 (L/K)	HC:TYR 33	GBRA1:THR 172	1.9 A	1x hb to GBRA1:THR 172	0.41	83.9%	33.7%
6D6T	1F4 (L/K)	HC:ARG 50	GBRA1:GLU 174	2.1 A	1x salt bridge to GBRA1:G	0.39	88.7%	98.1%
6D6T	1F4 (L/K)	LC:TYR 92	GBRA1:ALA 176	2.1 A	1x hb to GBRA1:ALA 176	0.85	82.5%	56.7%
6D6T	1F4 (L/K)	LC:TYR 92	GBRA1:VAL 198	2.4 A	1x hb to GBRA1:VAL 198	0.83	82.5%	90.1%
6D6T	1F4 (L/K)	LC:THR 31	GBRA1:ASP 199	2.1 A	1x hb to GBRA1:ASP 199	0.6	48.8%	64.7%
6D6T	1F4 (L/K)	LC:TYR 32	GBRA1:SER 200	1.9 A	1x hb to GBRA1:SER 200	0.67	100.0%	95.0%

Table S1. Protein interaction analysis in 6D6T and 6X3Z structures from PDB. They involve the specific interaction between the human α1β2γ2 GABA-A receptor and the monoclonal antibody (mAb) 1F4.

Protein Concentration measurement by UV absorbance

Protein concentrations were measured using a NanoDrop One^{C} Microvolume UV-Vis Spectrophotometer (Thermo Fisher Scientific, Waltham, MA, USA). Concentrations were estimated based on UV absorbance at 280 nm (A280 method), using 1–2 µL sample volumes. The "Other Protein" setting was selected from the menu, allowing manual input of custom molecular weight (M_w) and molar extinction coefficient (ε/1000) values. For (**1**) and 14b7* (**2**), molecular weights of 28.39 and 29.43 kDa and extinction coefficients of 51590 and 55600 $M^{-1}.cm^{-1}$, respectively, were used. PBS (0.01 M, pH 7.4) served as the blank.

A)

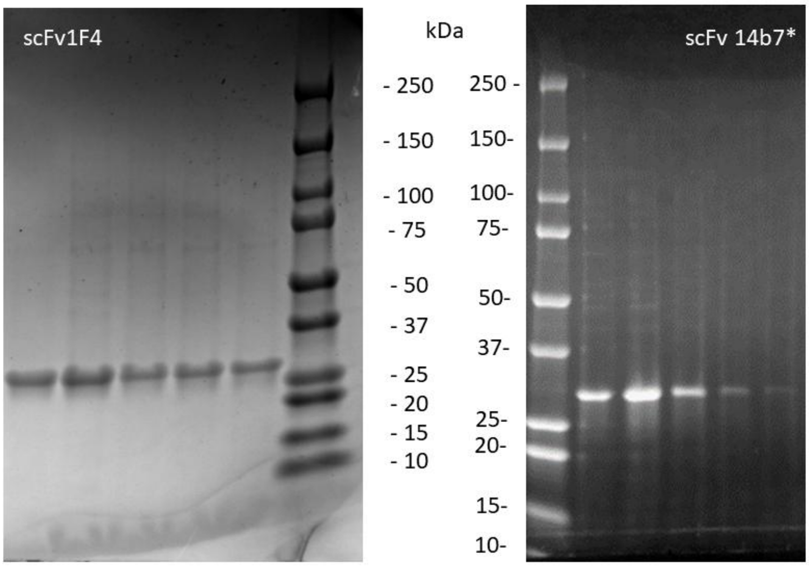

B)

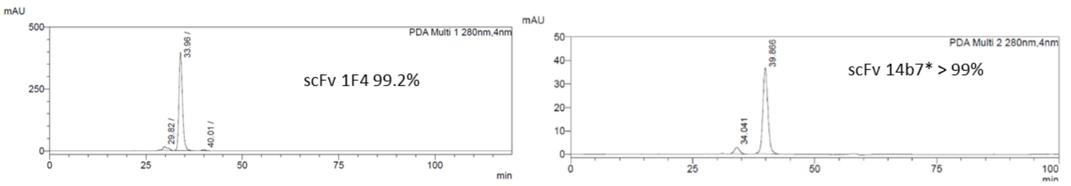

PDA Ch1 280nm

Peak#	Ret. Time	Area	Area%	Height	Height%
1	29.82	1416868	5.309	15187	3.659
2	33.96	25050762	93.864	396053	95.427
3	40.01	220692	0.827	3793	0.914
Total		26688321	100.000	415033	100.000

PDA Ch2 280nm

Peak#	Ret. Time	Area	Area%	Height	Height%
1	34.041	198972	7.069	2728	6.939
2	39.866	2615919	92.931	36592	93.061
Total		2814891	100.000	39321	100.000

Figure S1. Antibody fragments production. A) SDS-PAGE gel with different scFv1F4 (**1**) (left) or scFv 14b7* (**2**) (right) aliquots loaded in each well from the antibody fragment purification procedure. B) Size Exclusion Chromatography (SEC) of antibody fragments **1** and **2**.

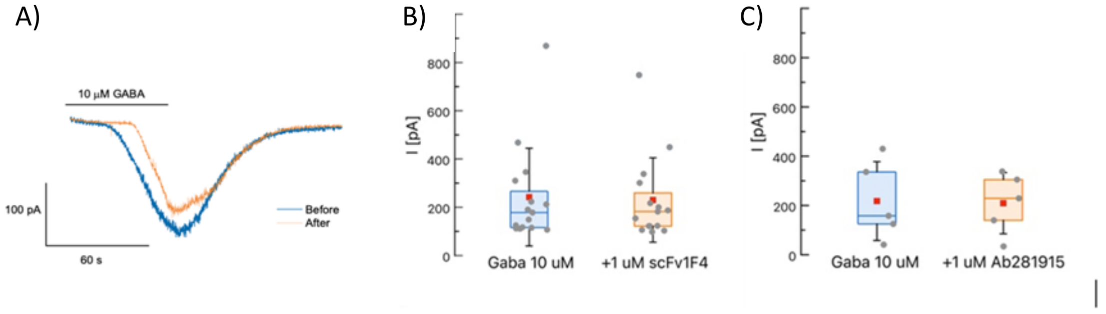

Figure S2. GABA-evoked current in WSS-1 cells. A) Representative current traces to submaximal 10 µM GABA before and after 1 µM scFv 1F4 (**1**) antibody treatment. B) Box plots of GABA-evoked current peak amplitudes under control conditions and after treatment with 1 µM antibody fragment **1** (n = 15) and C) Box plots of GABA-evoked current peak amplitudes under control conditions and after treatment with 1 µM commercial mAb 1F4 (Ab281915) (n = 5). Graphs made with Origin 2016 and LabPlot v2.11.1 (2025) https://labplot.org.

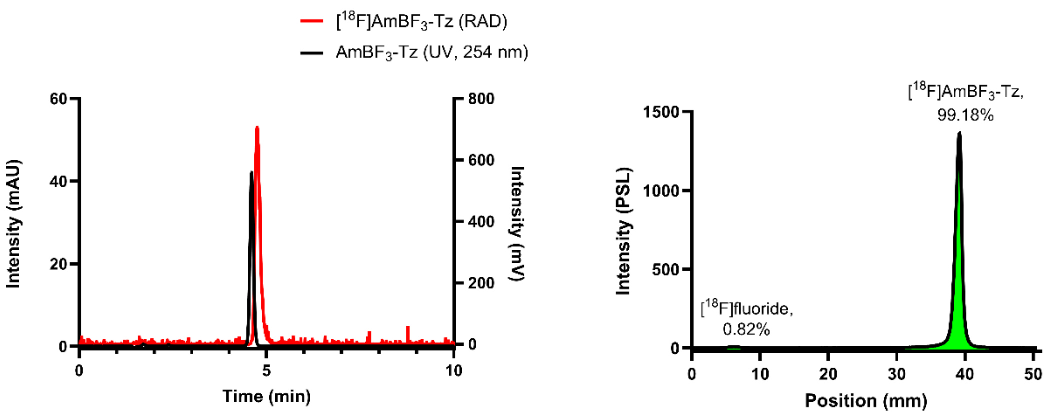

Figure S3. Quality control of the final purified [18F]AmBF$_3$-Tz ([18F]**3**). A) Radio-HPLC with representative UV chromatogram, [18F]**3** (R_t = 4.65 min); B) Radio-TLC: [18F]**3** (R_f = 0.75).

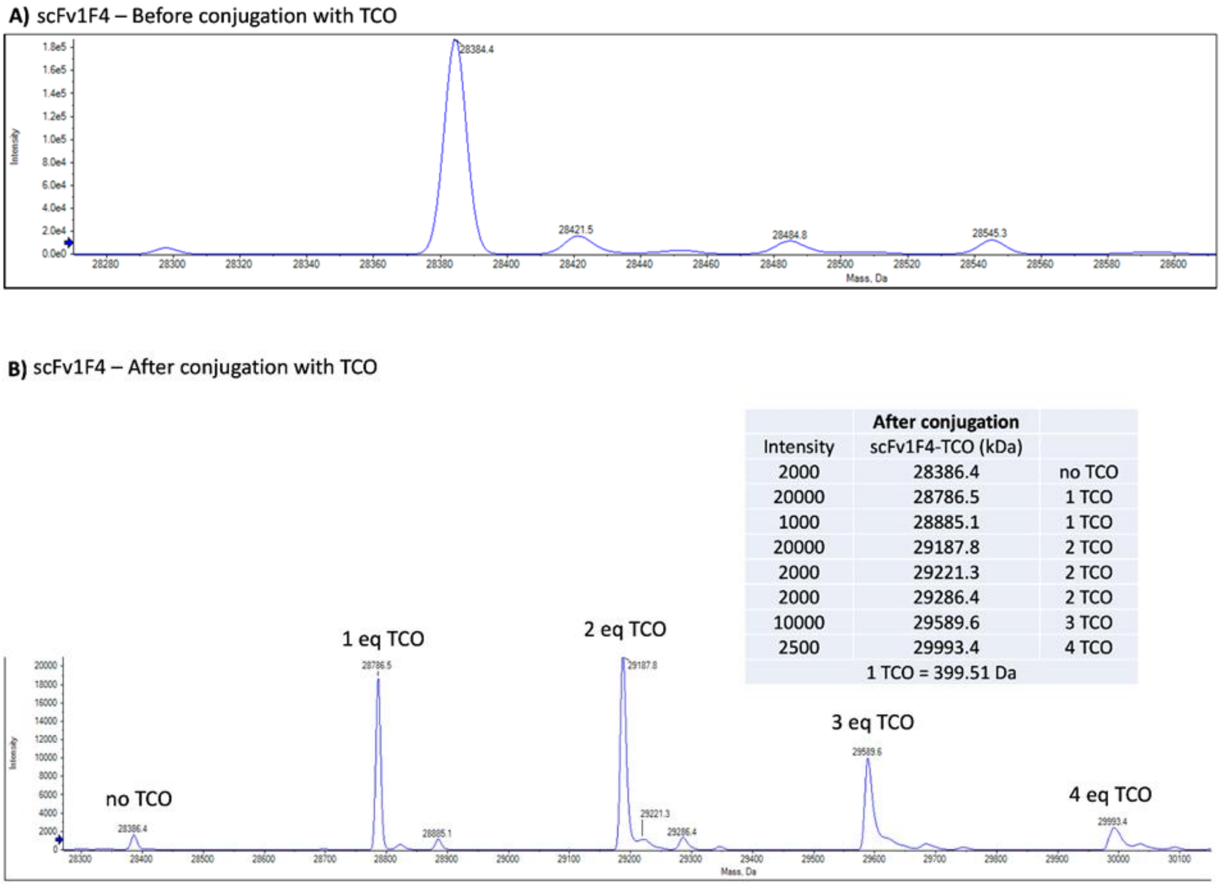

	After conjugation	
Intensity	scFv1F4-TCO (kDa)	
2000	28386.4	no TCO
20000	28786.5	1 TCO
1000	28885.1	1 TCO
20000	29187.8	2 TCO
2000	29221.3	2 TCO
2000	29286.4	2 TCO
10000	29589.6	3 TCO
2500	29993.4	4 TCO
	1 TCO = 399.51 Da	

Figure S4. **Figure S4**. Q-TOF mass spectrometry analysis of scFv 1F4 (**1**) and TCO-scFv 1F4 (**4**). A) Mass spectrum of scFv 1F4 prior to TCO conjugation. B) Mass spectrum of **1** after TCO conjugation.

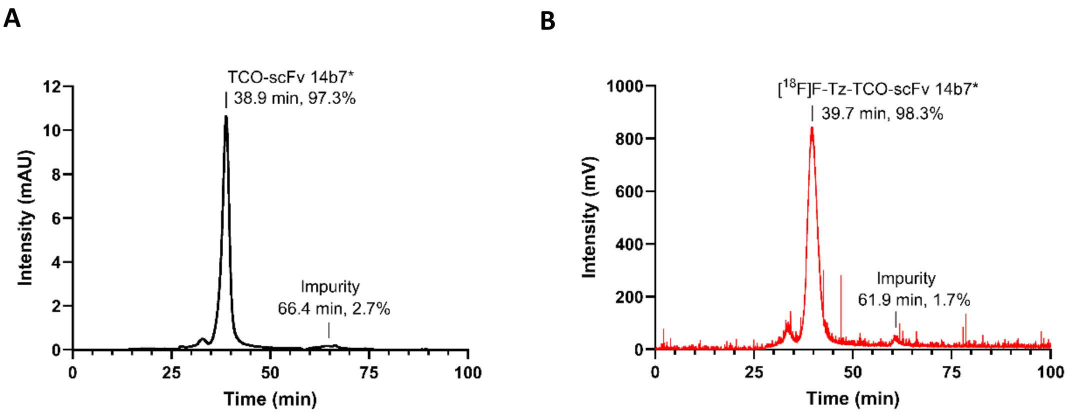

Figure S5. Quality control of TCO-scFv 14b7* (**5**) and [^{18}F]F-Tz-TCO-scFv 14b7* ([^{18}F]**5**). A) Representative UV SEC-HPLC chromatogram of **5** (280 nm), B) Representative SEC-HPLC radiochromatogram of purified [^{18}F]**5**.

%ID/g<br>Organs	[^{18}F]F-Tz-TCO-scFv 1F4 (n=4)	scFv 1F4 + [^{18}F]F-Tz-TCO-scFv 1F4 (n=4)	[^{18}F]F-Tz-TCO-scFv 14b7* (n=4)
Blood	10.70±1.93	7.29±0.26*	3.30±0.24***/***
Plasma	20.04±3.44	13.32±0.54**	6.19±0.31***/***
Blood cells	4.17±0.79	0.80±0.10***	0.34±0.04***/***
Spleen	2.83±0.42	2.03±0.09**	1.98±0.18**
Pancreas	1.32±0.10	1.11±0.10*	0.74±0.23**/*
Heart	2.48±0.59	1.37±0.32*	0.77±0.06**/**
Kidneys	23.19±6.88	12.85±1.50*	16.16±2.43
Bladder (urine)	112.89±46.53	82.59±9.44	222.38±136.16
Gonads	1.72±1.22	0.42±0.02	1.23±0.71
Lungs	2.88±0.90	1.50±0.64*	0.84±0.47**
Harderian glands	1.54±0.38	1.03±0.15*	0.57±0.04**/***
Brain	0.11±0.05	0.04±0.02*	0.02±0.01**
Gallbladder	10.07±1.51	34.4±37.15	17.84±12.98
Liver	6.60±1.45	4.20±0.33*	4.71±0.19*/*
Stomach	1.49±1.32	0.43±0.11	0.74±0.53
Small intestine	5.50±2.35	14.43±2.00*	8.39±1.25 /**
Large intestine	6.07±1.48	1.12±0.21*	1.23±0.82**

Table S2. *Ex vivo* biodistribution of [^{18}F]F-Tz-TCO-scFv 1F4 ([^{18}F]**4**) (baseline and blocked), and [^{18}F]F-Tz-TCO-scFv 14b7* ([^{18}F]**5**). Radioactivity (%ID/g) in organs at 2 h after i.v. injection of the tracer. Under blocking conditions, scFv 1F4 (**1**) (50 ug) was injected 60 min before radiotracer administration. Statistical analysis was performed with the [^{18}F]**4** group as the reference (*$P < 0.05$, **$P < 0.01$, ***$P < 0.001$).

%ID/g Organs	[18F]F-Tz-TCO-scFv 1F4 $_{\text{high molar activity}}$ (n=8)	[18F]F-Tz-TCO-scFv 1F4 $_{\text{low molar activity}}$ (n=6)
Blood	4.06±0.46	2.17±0.75***
Plasma	7.74±0.84	4.10±1.41***
Blood cells	0.42±0.06	0.23±0.06***
Spleen	1.29±0.18	0.94±0.12**
Pancreas	0.58±0.12	0.40±0.14*
Heart	0.92±0.22	0.56±0.19**
Kidneys	6.15±2.40	5.64±2.65
Bladder (urine)	55.36±25.16	26.14±18.41*
Gonads	0.64±0.76	1.12±0.93
Lungs	1.16±0.37	0.73±0.36*
Harderian glands	0.67±0.15	0.45±0.11**
Brain	0.03±0.01	0.02±0.01**
Gallbladder	18.16±4.43 (n=7)	32.39±11.39*
Liver	3.27±0.71	3.26±0.87
Stomach	1.04±0.50	0.70±0.35
Small intestine	6.80±4.42	5.68±2.97
Large intestine	13.80±3.77	18.49±6.04

Table S3. *Ex vivo* biodistribution of [^{18}F]F-Tz-TCO-scFv 1F4 ([^{18}F]**4**) after i.v. injection of the tracer with high (injected dose 6.62 ± 2.90 µg, n = 8) or low molar activity (A_m) (injected dose 12.08 ± 2.37 µg, n = 6). Radioactivity (%ID/g) in different organs at 4 h after i.v. injection of the tracer (*P < 0.05, **P < 0.01, ***P < 0.001).

Organs \ %ID/g	TCO-scFv 1F4 + [18F]AmBF3-Tz (60 min) (n=8)	[18F]AmBF3-Tz (60 min) (n=4)	TCO-scFv 1F4 + [18F]AmBF3-Tz (120 min) (n=8)	[18F]AmBF3-Tz (120 min) (n=4)
Blood	2.14±0.55	0.93±0.06**	1.04±0.37	0.52±0.11*
Plasma	3.15±0.89	0.62±0.17***	1.31±0.54	0.31±0.08**
Blood cells	1.23±0.34	1.26±0.18	0.77±0.22	0.78±0.20
Spleen	1.15±0.33 (n=4)	1.15±0.32	0.53±0.16	0.49±0.09
Pancreas	1.50±0.20 (n=4)	1.68±0.64	0.68±0.16	0.70±0.15
Heart	0.73±0.24	0.53±0.04	0.36±0.13	0.28±0.52
Kidneys	4.60±0.89	2.22±0.58***	1.25±0.48	1.07±0.31
Bladder (urine)	103.26±72.42	272.69±86.21**	31.49±29.26	163.09±158.86*
Gonads	1.31±1.48	0.58±0.28	0.77±0.80	0.27±0.18
Lungs	1.23±0.75	0.80±0.23	0.38±0.17	0.26±0.10
Harderian glands	1.52±0.33	1.39±0.93	0.47±0.10	0.51±0.12
Brain	0.03±0.01	0.03±0.02	0.01±0.00	0.01±0.00
Gallbladder	6.98±2.84 (n=3)	5.26±1.74	4.32±2.30	6.12±7.98
Liver	3.69±0.88	3.58±0.97	0.85±0.29	0.48±0.12*
Stomach	1.14±0.54	0.64±0.47	0.33±0.48	0.16±0.07
Small intestine	5.53±1.53	6.02±1.47	2.66±1.76	5.46±3.88
Large intestine	1.84±0.80	1.93±0.22	3.84±2.32	4.82±2.01

Table S4. *Ex vivo* biodistribution after pretargeting α1 subunit of GABA-A receptors with TCO-scFv 1F4 (**4**) (10 μg i.v.) 60 min prior to [18F]AmBF$_3$-Tz ([18F]**3**). Radioactivity (%ID/g) in different organs at 60 and 120 min after i.v. injection of [18F]**3** (*P < 0.05, **P < 0.01, ***P < 0.001).

NMR-spectra of $AmBF_3$-Tz (3)

^{1}H-NMR

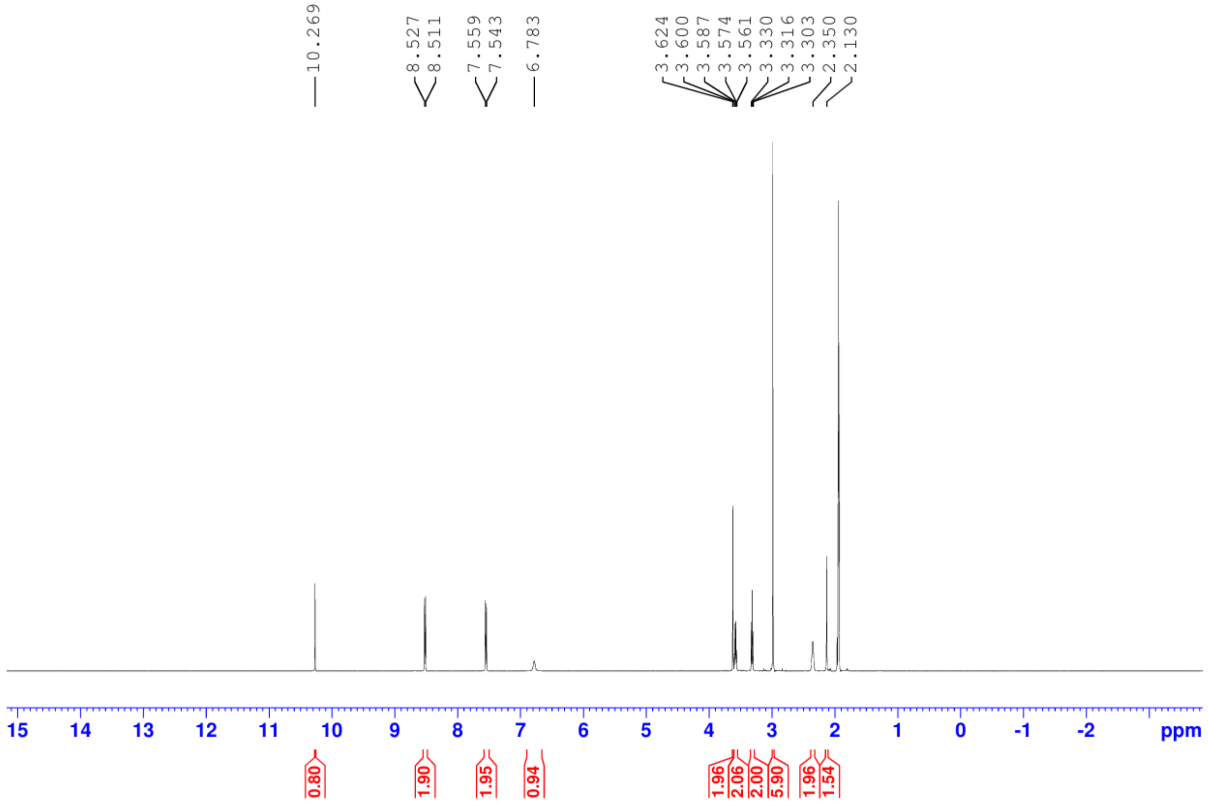

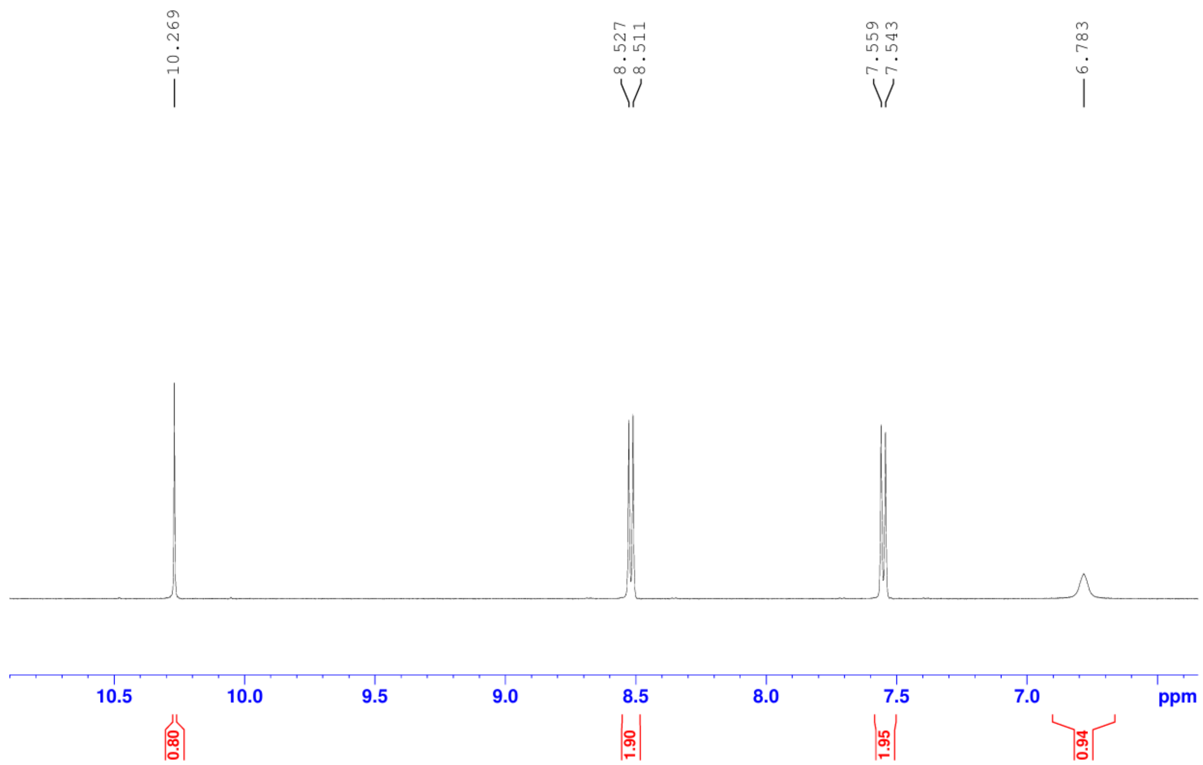
10.269
8.527
8.511
7.559
7.543
6.783
10.5
10.0
9.5
9.0
8.5
8.0
7.5
7.0
ppm
0.80
1.90
1.95
0.94

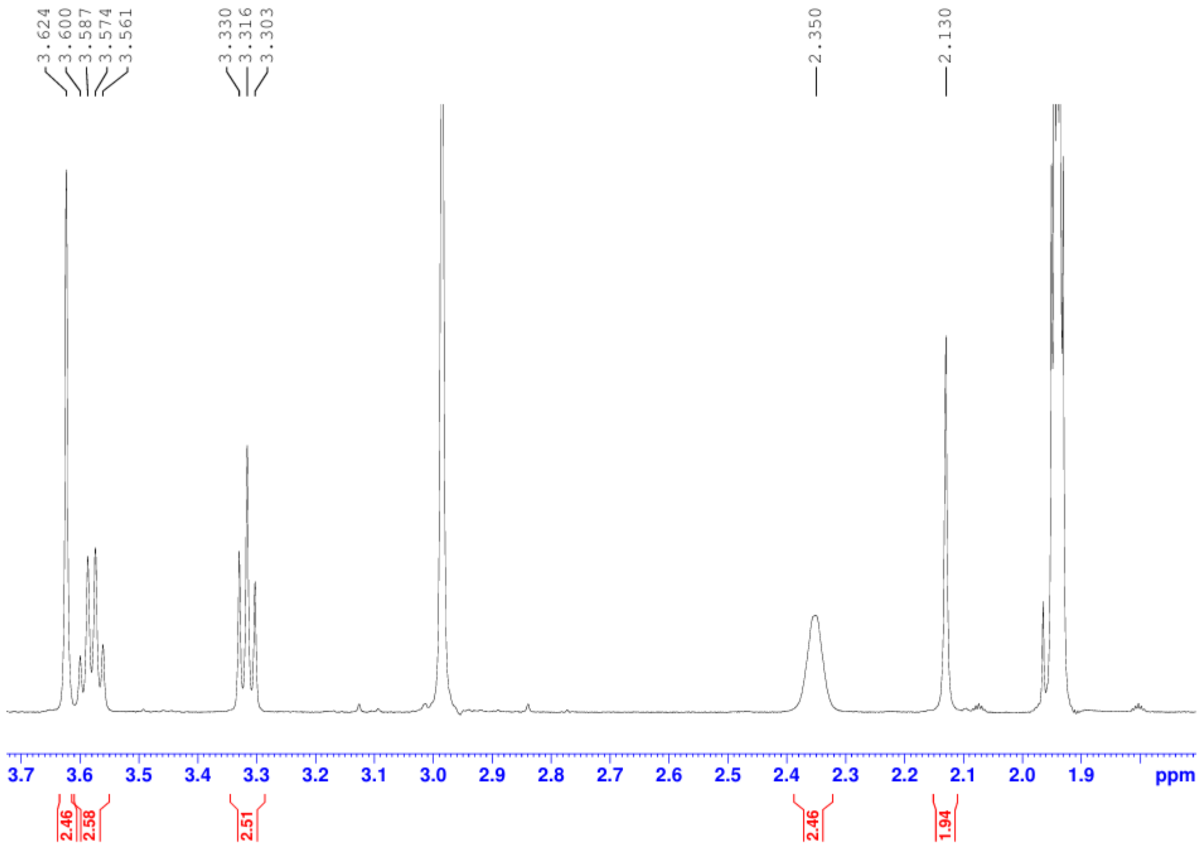
3.624
3.600
3.587
3.574
3.561
3.330
3.316
3.303
2.350
2.130
3.7
3.6
3.5
3.4
3.3
3.2
3.1
3.0
2.9
2.8
2.7
2.6
2.5
2.4
2.3
2.2
2.1
2.0
1.9
ppm
2.46
2.58
2.51
2.46
1.94

^{19}F-NMR

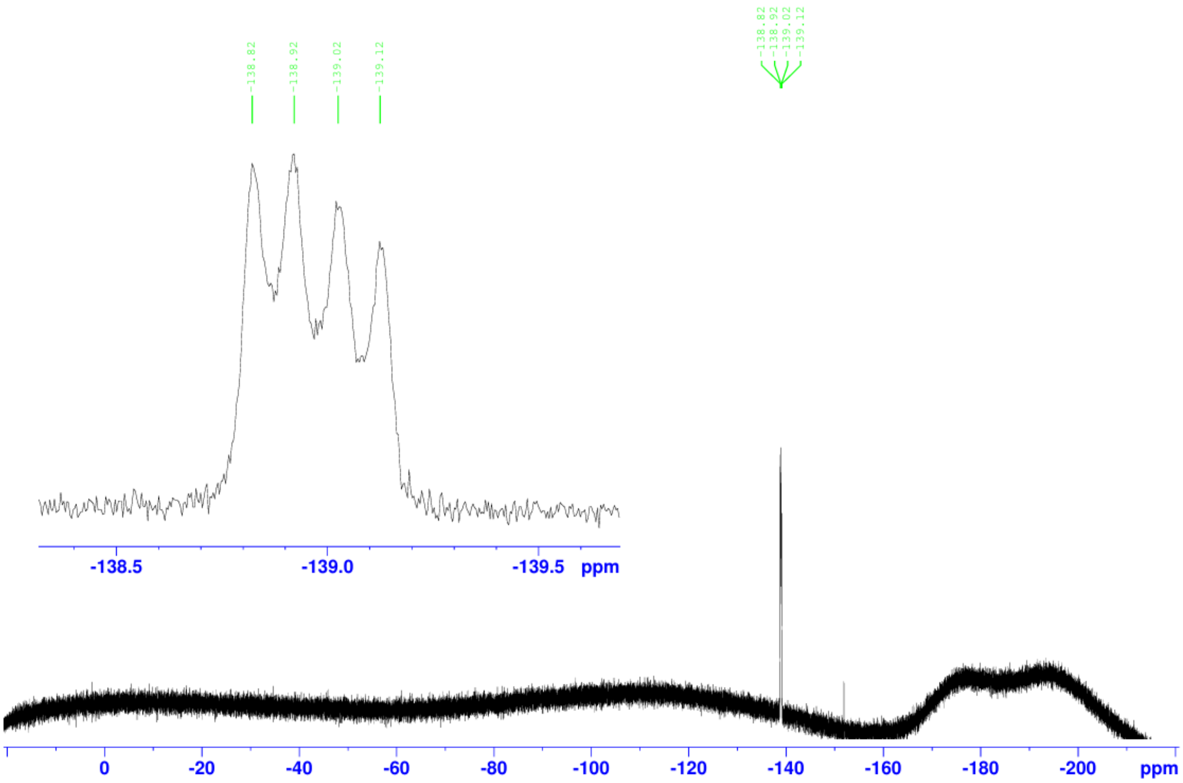

-138.82
-138.92
-139.02
-139.12
-138.5
-139.0
-139.5
ppm
0
-20
-40
-60
-80
-100
-120
-140
-160
-180
-200
ppm

^{13}C-NMR

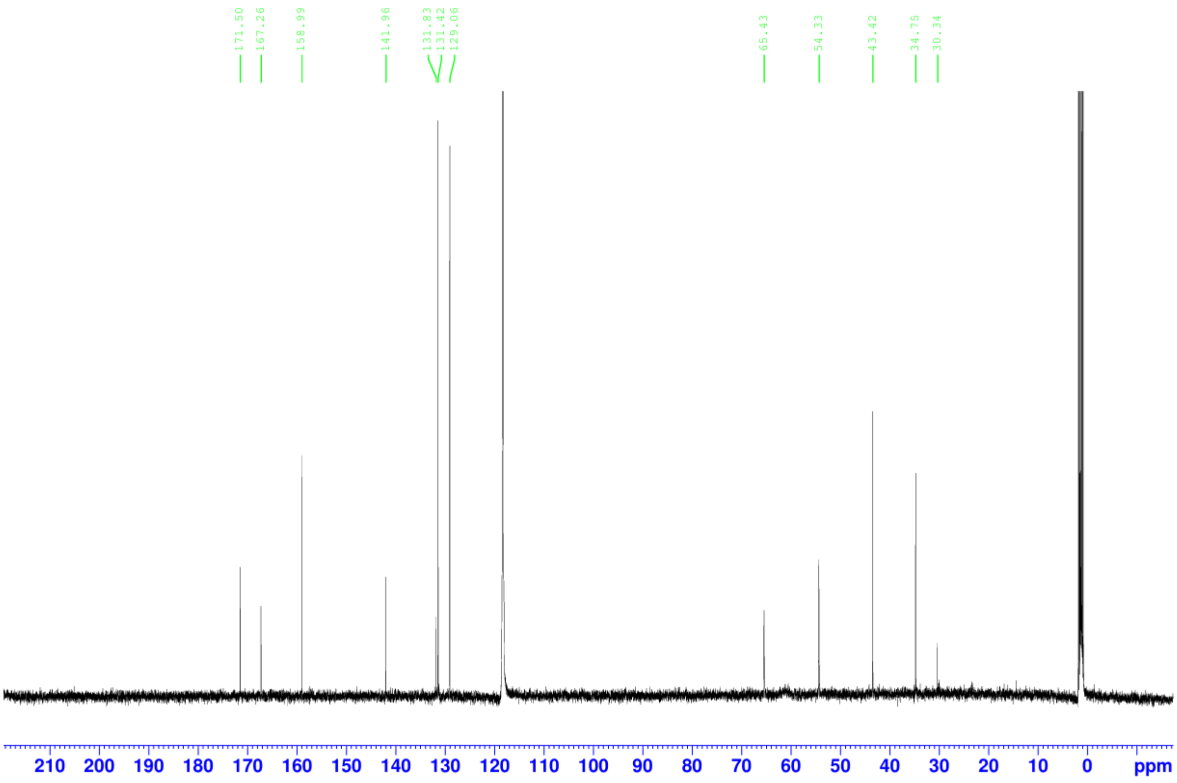

171.50
167.26
158.99
141.96
131.83
131.42
129.06
65.43
54.33
43.42
34.75
30.34
210 200 190 180 170 160 150 140 130 120 110 100 90 80 70 60 50 40 30 20 10 0 ppm

COSY

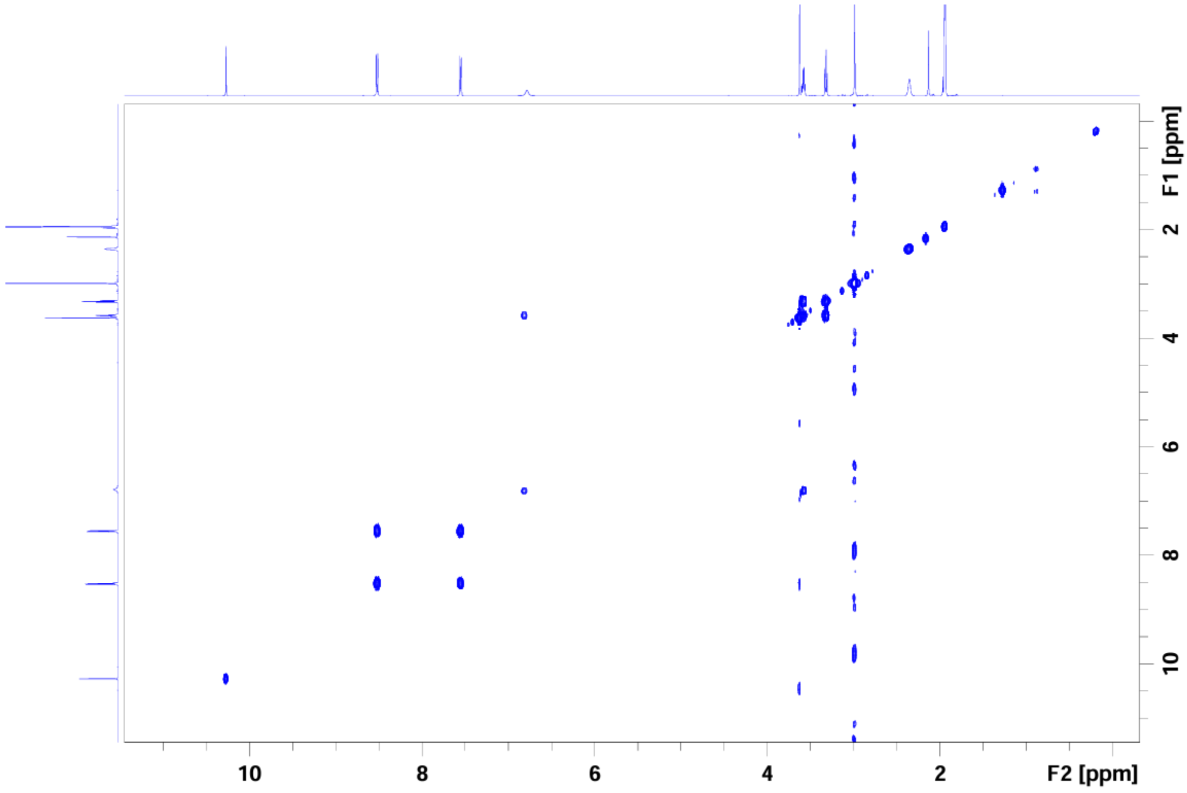

F1 [ppm]
2
4
6
8
10
10
8
6
4
2
F2 [ppm]

HSQC

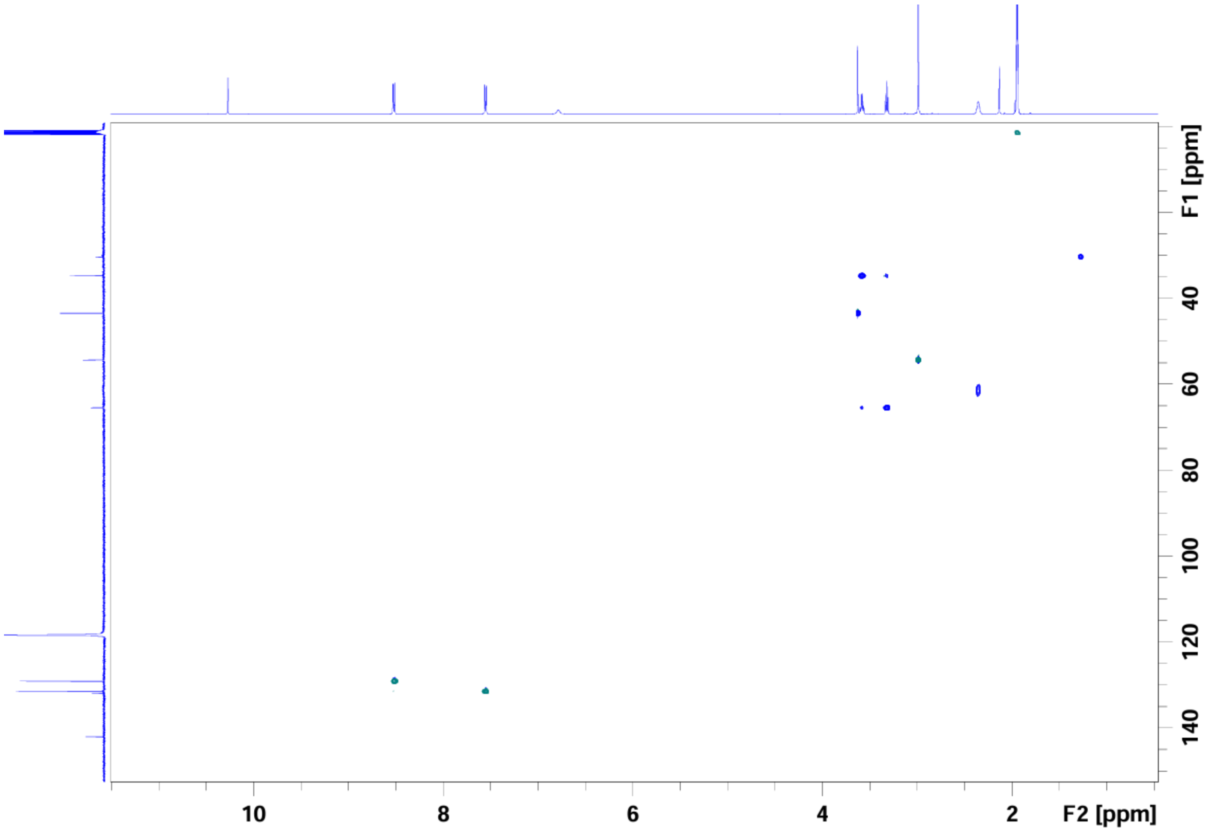

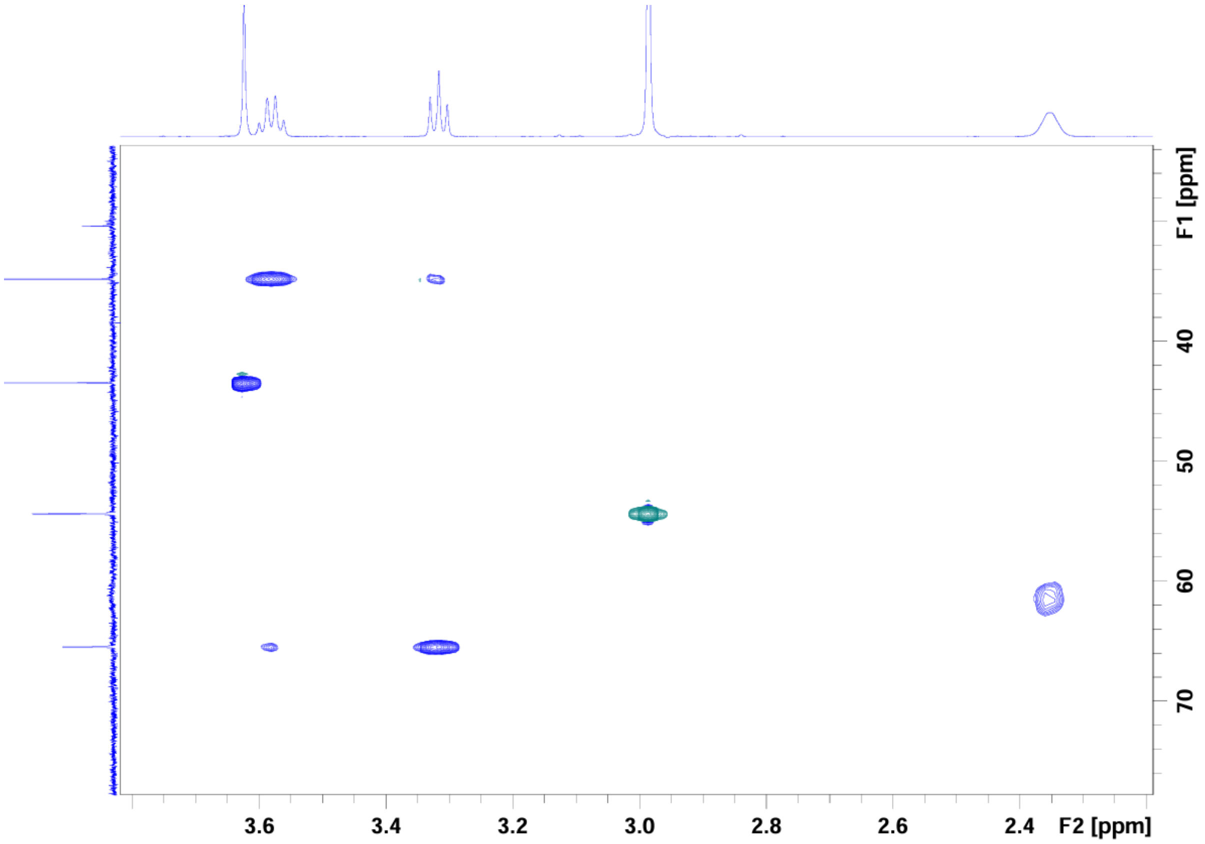

HMBC

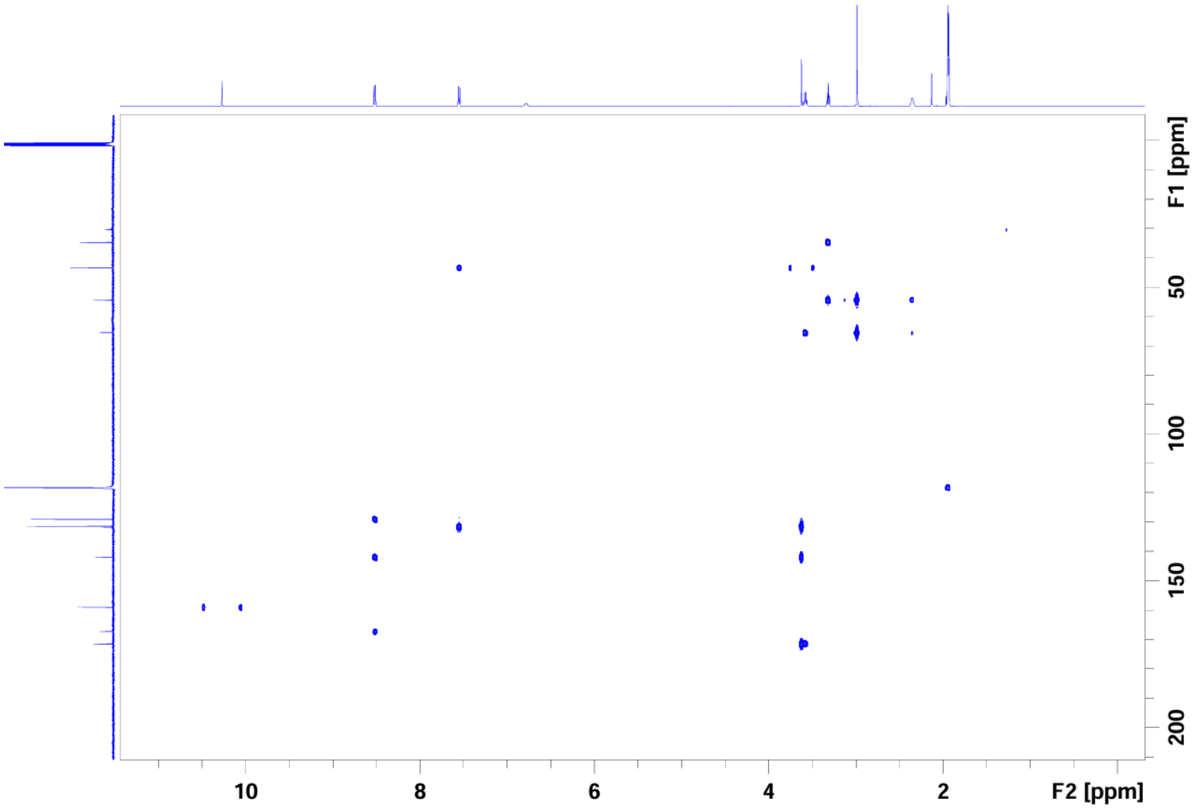

F1 [ppm]
50
100
150
200
10
8
6
4
2
F2 [ppm]

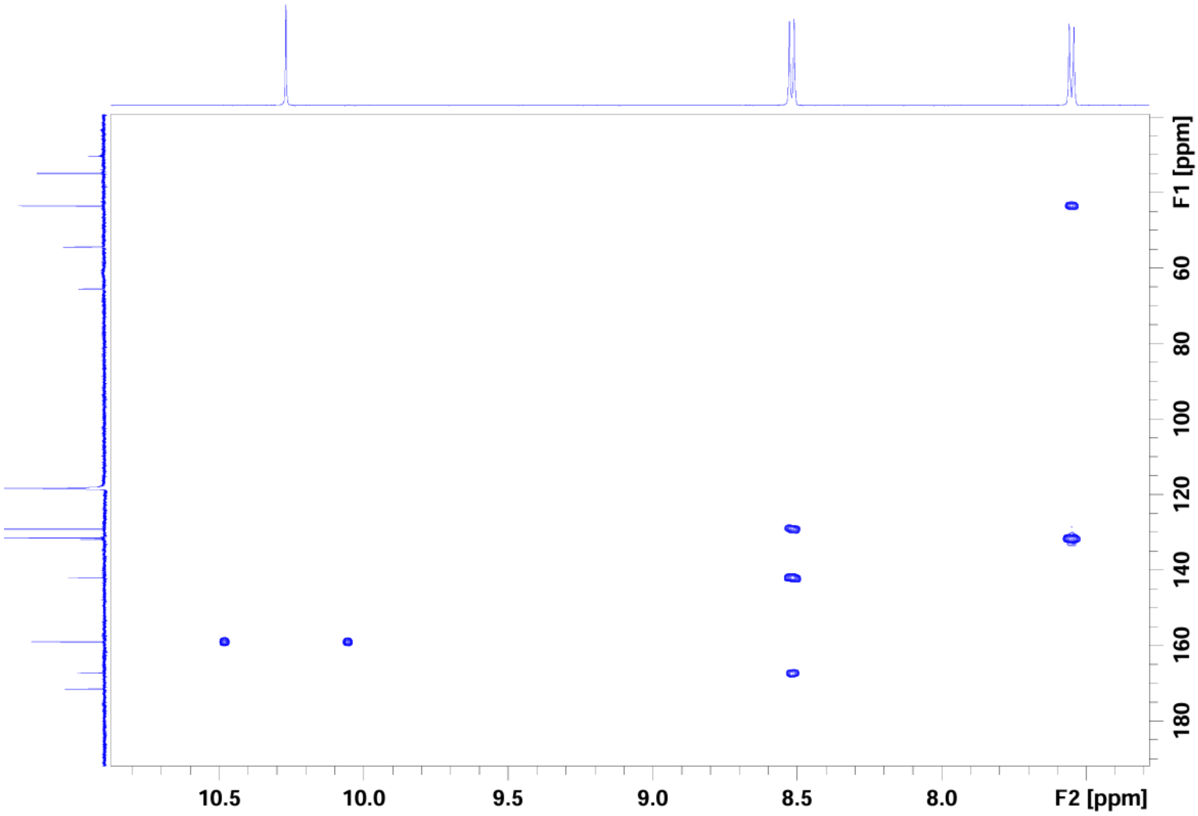

F1 [ppm]
60
80
100
120
140
160
180
10.5
10.0
9.5
9.0
8.5
8.0
F2 [ppm]

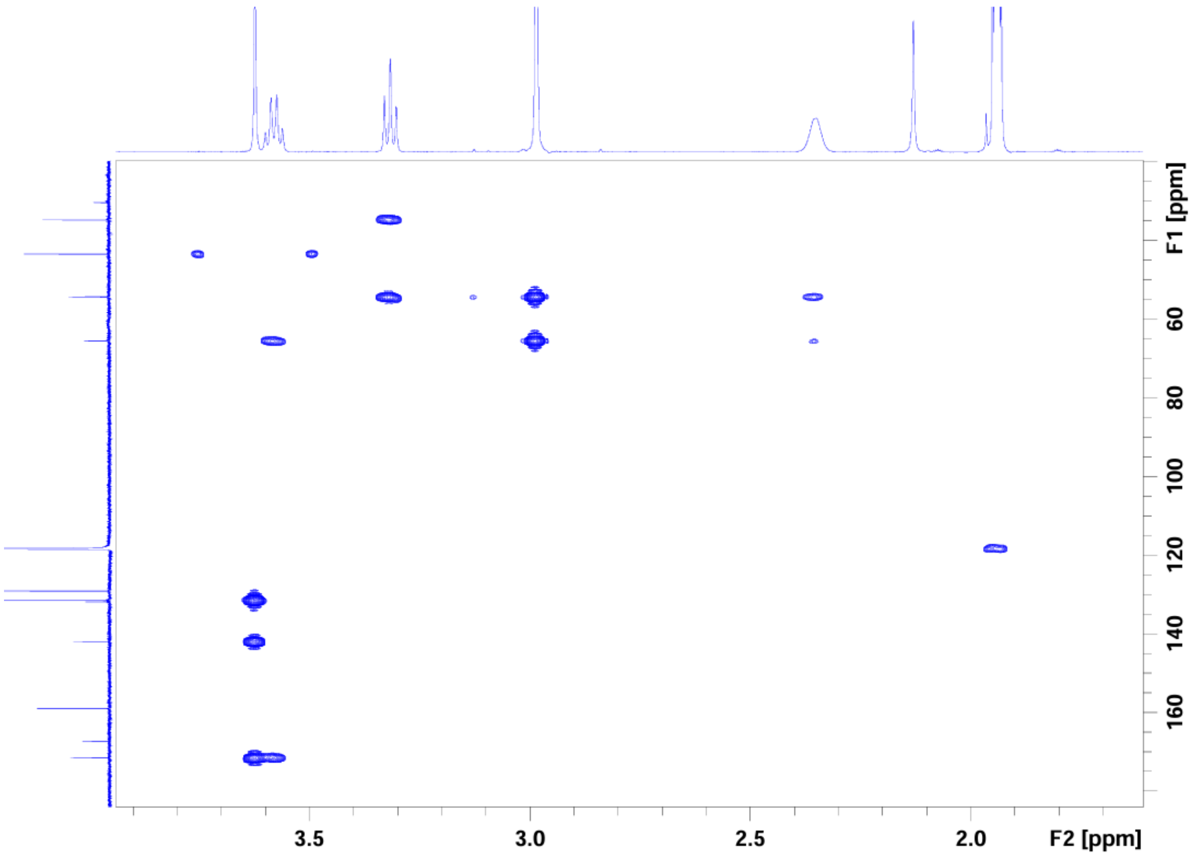
F1 [ppm]
60
80
100
120
140
160
3.5
3.0
2.5
2.0
F2 [ppm]
